# First report of spotted fever group *Rickettsia aeschlimannii* in *Hyalomma turanicum*, *Haemaphysalis bispinosa*, and *Haemaphysalis montgomeryi* infesting domestic animals: updates on the epidemiology of tick-borne *Rickettsia aeschlimannii*

**DOI:** 10.3389/fmicb.2023.1283814

**Published:** 2023-12-15

**Authors:** Abdul Majid, Mashal M. Almutairi, Abdulaziz Alouffi, Tetsuya Tanaka, Tsai-Ying Yen, Kun-Hsien Tsai, Abid Ali

**Affiliations:** ^1^Department of Zoology, Abdul Wali Khan University Mardan, Mardan, Pakistan; ^2^Department of Pharmacology and Toxicology, College of Pharmacy, King Saud University, Riyadh, Saudi Arabia; ^3^King Abdulaziz City for Science and Technology, Riyadh, Saudi Arabia; ^4^Laboratory of Infectious Diseases, Joint Faculty of Veterinary Medicine, Kagoshima University, Kagoshima, Japan; ^5^Department of Public Health, Institute of Environmental and Occupational Health Sciences, College of Public Health, National Taiwan University, Taipei, Taiwan

**Keywords:** ticks, *Rickettsia aeschlimannii*, *Hyalomma turanicum*, *Haemaphysalis bispinosa*, *Haemaphysalis montgomeryi*

## Abstract

Tick-borne *Rickettsia* spp. have long been known as causative agents for zoonotic diseases. We have previously characterized *Rickettsia* spp. in different ticks infesting a broad range of hosts in Pakistan; however, knowledge regarding *Rickettsia aeschlimannii* in *Haemaphysalis* and *Hyalomma* ticks is missing. This study aimed to obtain a better understanding about *R. aeschlimannii* in Pakistan and update the knowledge about its worldwide epidemiology. Among 369 examined domestic animals, 247 (66%) were infested by 872 ticks. Collected ticks were morphologically delineated into three genera, namely, *Rhipicephalus*, *Hyalomma,* and *Haemaphysalis*. Adult females were the most prevalent (number ₌ 376, 43.1%), followed by nymphs (303, 34.74%) and males (193, 22.13%). Overall, genomic DNA samples of 223 tick were isolated and screened for *Rickettsia* spp. by the amplification of rickettsial *gltA*, *ompA*, and *ompB* partial genes using conventional PCR. Rickettsial DNA was detected in 8 of 223 (3.58%) ticks including nymphs (5 of 122, 4.0%) and adult females (3 of 86, 3.48%). The rickettsial *gltA*, *ompA*, and *ompB* sequences were detected in *Hyalomma turanicum* (2 nymphs and 1 adult female), *Haemaphysalis bispinosa* (1 nymph and 1 adult female), and *Haemaphysalis montgomeryi* (2 nymphs and 1 adult female). These rickettsial sequences showed 99.71–100% identity with *R. aeschlimannii* and phylogenetically clustered with the same species. None of the tested *Rhipicephalus microplus*, *Hyalomma isaaci*, *Hyalomma scupense*, *Rhipicephalus turanicus*, *Hyalomma anatolicum*, *Rhipicephalus haemaphysaloides*, *Rhipicephalus sanguineus, Haemaphysalis cornupunctata*, and *Haemaphysalis sulcata* ticks were found positive for rickettsial DNA. Comprehensive surveillance studies should be adopted to update the knowledge regarding tick-borne zoonotic *Rickettsia* species, evaluate their risks to humans and livestock, and investigate the unexamined cases of illness after tick bite among livestock holders in the country.

## Introduction

Ticks are important ectoparasites due to their capacity to feed on animals including humans (De la Fuente et al., 2017) and transmit various pathogens including viruses, bacteria, and protozoans to their hosts (Boulanger et al., 2019). Among bacteria, *Rickettsia* species are transmitted by ticks, mites, and fleas, causing rickettsioses (Fournier and Raoult, 2009; Labruna, 2009). The main tick genera that are the potential vectors for *Rickettsia* spp. are *Amblyomma*, *Dermacentor*, *Ixodes*, *Hyalomma*, *Rhipicephalus,* and *Haemaphysalis* (Fournier and Raoult, 2009). Several studies have revealed the extensive diversity of the spotted fever group rickettsiae in different tick species and geographic locations (Socolovschi et al., 2009). Approximately 34 species in the genus *Rickettsia* have been validated, and several others are yet to be determined to the species level (El Karkouri et al., 2022). Spotted fever group *Rickettsia* spp. are distributed in Pakistan and have recently been reported in different ticks including *Rh. microplus, Rh. haemaphysaloides, Rh. turanicus, H. kashmirensis,* and *H. cornupunctata* infesting different animals (Ali et al., 2021, 2022; Khan et al., 2023).

Hard ticks are important reservoirs for various *Rickettsia* species including *Rickettsia aeschlimannii* (Parola et al., 2013). Previously, *R. aeschlimannii* has been detected in different tick species belonging to different genera such as *Rhipicephalus, Hyalomma, Amblyomma*, *Haemaphysalis*, and *Ixodes* in Morocco, Spain, Senegal, and Bolivia (Beati et al., 1997; Fernández-Soto et al., 2003; Mediannikov et al., 2010; Tomassone et al., 2010). The first human infection by *R. aeschlimannii* was documented serologically in Morocco (Raoult et al., 2002). Till then, several cases of human infection with tick-borne *R. aeschlimannii* have been reported (Raoult et al., 2002; Znazen et al., 2006; Mokrani et al., 2008; Germanakis et al., 2013; Igolkina et al., 2022). Molecular identification and genetic characterization of *Rickettsia* spp. are based on the *gltA* (citrate synthase gene), *ompA* (outer membrane protein A), *ompB* (outer membrane protein B), and *sca*4 (surface cell antigen 4) genes (Roux et al., 1997; Fournier et al., 2003).

Earlier, we characterized pathogenic and undetermined *Rickettsia* spp. associated with different ticks parasitizing a wide range of vertebrate hosts in Pakistan (Karim et al., 2017; Ali et al., 2021, 2022, 2023; Numan et al., 2022; Aneela et al., 2023; Shehla et al., 2023; Ullah et al., 2023). However, there is a paucity of information regarding the presence of *R. aeschlimannii* in different ticks in the country, and its potential risks to the public and animal’s health. Updated knowledge regarding the epidemiology, association with ticks infesting different hosts, and phylogenetic position of spotted fever group (SFG) *R. aeschlimannii* is essential for effective management of infection. This study aimed to molecularly characterize tick-borne *Rickettsia* spp. in selected districts of Pakistan and update the information about the global epidemiology of uncharacterized *Rickettsia* spp.

## Materials and methods

### Study area

**Figure 1:**
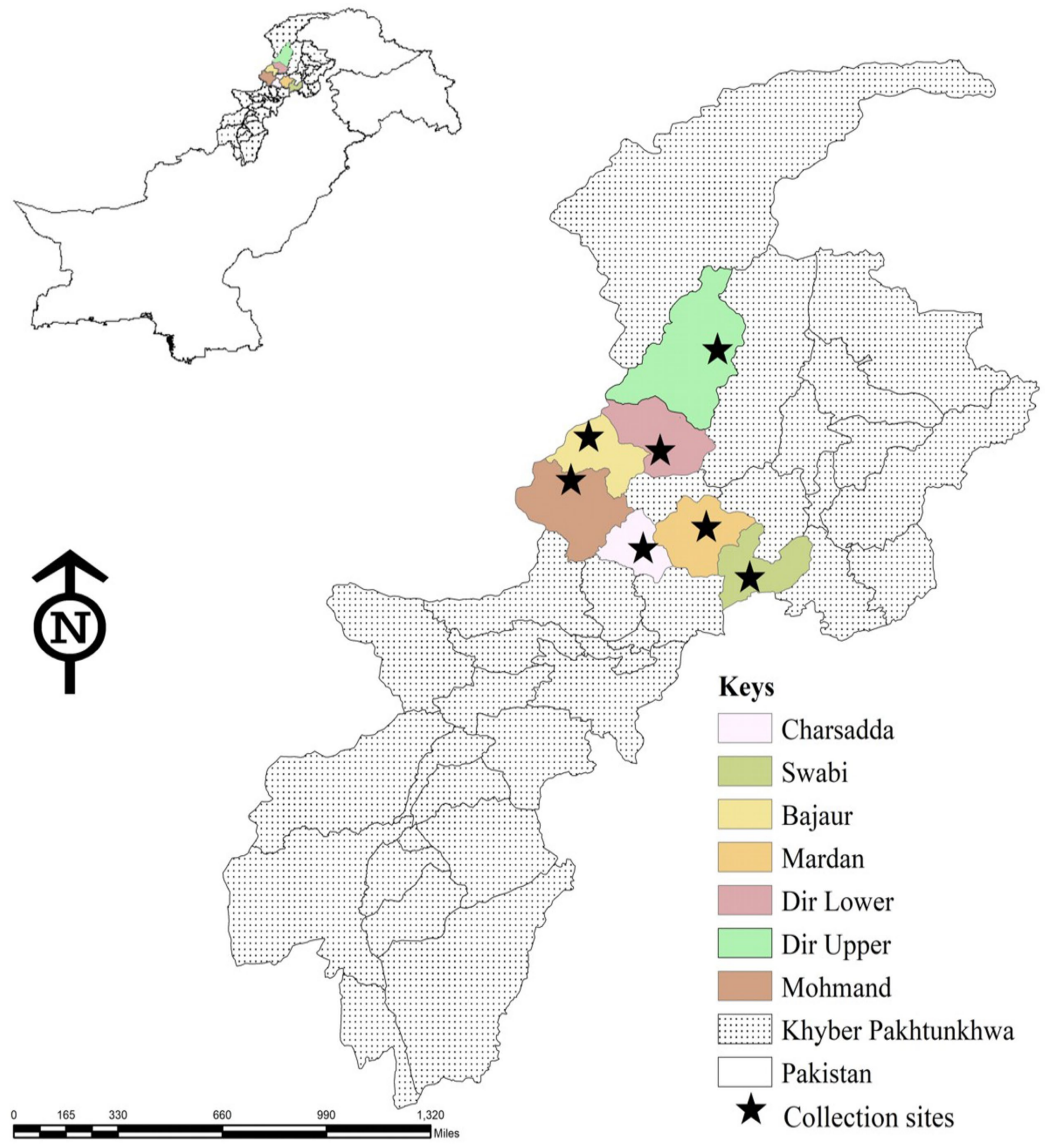
Map showing locations in seven districts of Khyber Pakhtunkhwa, where ticks were collected from domestic animals.

Tick specimens were collected from seven districts of Khyber Pakhtunkhwa (KP), a province in northwest Pakistan, namely, Mohmand (34.5356°N, 71.2874°E), Bajaur (34.7865°N, 71.5249° E), Swabi (34.0719°N, 72.4732°E), Charsadda (34.1682°N, 71.7504°E), Mardan (34.1986°N, 72.0404°E), Dir-Upper (35.3356°N, 72.0468°E), and Dir-Lower (34.9161°N, 71.8097°E). Data regarding the host, ticks, climate, and location were noted. Climatic information was taken from the website climate-data.org. Geo-coordinates of location sites were obtained from the global positioning system and were entered into a spreadsheet, and the study map was designed in ArcGIS10.8.1.3 (ESRI, Redlands, CA, USA) (Figure 1).

### Ethical statement

The design of the current study has received approval from the members of the Advanced Study and Research Board (Dir/A&R/AWKUM/2023/0014) and the Faculty of Zoology Department, Abdul Wali Khan University Mardan, Pakistan. Permission was obtained from the owner of the animal before collecting ticks from their animals.

### Collection and morphological identification of ticks

Tick specimens were collected conveniently from March 2020 to February 2021 from domestic animals in seven districts of Khyber Pakhtunkhwa, Pakistan. The collection was opportunistic occurring whenever tick-infested animals were found within the survey regions. The whole body of the animal was examined for ticks. With the help of curved forceps, ticks were collected carefully so that the morphological features of ticks were not damaged. Every sample was tagged with its location of collection, date, and species of the animal.

The collected tick specimens were morphologically identified through a stereo microscope (CM100, China) by using standard taxonomic keys (Hoogstraal, 1962, 1966, 1973; Walker et al., 2000; Apanaskevich et al., 2008; 2010; Geevarghese and Mishra, 2011; Ali et al., 2022).

### Genomic DNA extraction

All ticks were identified morphologically, and genomic DNA was individually extracted from a subset of 223 (122N, 86F, and 15M) ticks. Ticks were cleaned with 70% ethanol, followed by distilled water and phosphate-buffered saline for the elimination of surface contaminants. Each rinsed tick was separately kept in a 1.5 mL tube and subjected to drying within an incubator for 30 min. Using a sterilized scalpel, the samples were cut into pieces inside the Eppendorf tube. The phenol-chloroform method was used for the extraction of genomic DNA from the ticks (Sambrook et al., 1989). The DNA concentration in each extracted sample was quantified with Nanodrop (OPTIZEN, Daejeon, South Korea).

### Amplification of targeted rickettsial DNA

The extracted DNA was used in conventional PCR (Thermo Fisher Scientific, and Walham, MA, USA), to amplify fragments of three genes of *Rickettsia,* namely, *gltA* (citrate synthase gene), *ompA* (outer membrane protein A), and *ompB* (outer membrane protein B). PCR was conducted in a 25 μL reaction mixture, containing 1 μL of each primer (forward and reverse primers), 8.5 μL of PCR water, 2 μL of template DNA, and 12.5 μL of DreamTaq PCR Master Mix (2×) (Thermo Scientific, Waltham, MA, USA). The primers used in the current study are presented in Table 1, and thermocycling conditions were set as previously used (Regnery et al., 1991; Roux and Raoult 2000; Labruna et al., 2004). Positive and negative control samples were *Rickettsia massiliae* DNA and “nuclease-free” water, respectively (Shehla et al., 2023). The amplified products of each PCR were observed by electrophoresis on 2% agarose gel and stained with ethidium-bromide, and the results were visualized on the Gel Doc system (BioDoc-It™ Imaging Systems, Upland, CA, USA).

**Table 1:** Primers used for the amplification of rickettsial DNA in the current study.

Gene	Primer	Sequence(5′-3′)	Amplicon size	Annealing Temperatures	Reference
*gltA*	CS-78	GCAAGTATCGGTGTGAGGATGTAAT	401 bp	56°C	Labruna et al. (2004)
	CS-323	GCTTCCTTAAAATTCAATAAATCAGGAT			
*ompA*	Rr190.70p	ATGGCGAATATTTCTCCAAAA	532 bp	55°C	Regnery et al. (1991)
	Rr190.602n	AGTGCAGCATTCGCTCCCCCT			
*ompB*	120-M59	CCGCAGGGTTGGTAACTGC	862 bp	50°C	Roux and Raoult (2000)
	120–807	CCTTTTAGATTACCGCCTAA			

### Sequencing and phylogenetic analysis

The amplified DNA fragments were purified using the GENECLEAN II Kit (Qbiogene, Illkirch, France) and sequenced using the Sanger sequencing (Macrogen, Inc., Seoul, South Korea) in both forward and reverse directions. Poor-quality sequences were removed by trimming all the obtained sequences using SeqMan v 5.00 (DNASTAR, Inc.), and a consensus sequence was generated. Sequences with the highest identity were selected from GenBank using the Basic Local Alignment Search Tool (BLAST) on the user interface of National Center for Biotechnology Information, and these sequences were aligned with the obtained sequences by BioEdit v. 7.0.5 using CLUSTALW multiple alignments (Thompson et al., 1994), followed by MUSCLE alignment (Edgar, 2004). The Neighbor-Joining method was applied for obtaining phylogenies with 1,000 bootstrap replicates in Molecular Evolutionary Genetic Analysis (MEGA-X) software (Kumar et al., 2018). The sequences that were obtained made up the final positions in the dataset.

### Data analysis

The recorded data of tick-infested hosts and tick distribution were described with frequency and percentage using descriptive statistics. Fisher’s exact test was used to determine the association between host, tick species, rickettsial species, and locations in GraphPad Prism software (V 5.0). *p*-value <0.05 was considered as significant standard.

### Literature-based search

The literature-based search was carried out by using different databases including ScienceDirect, PubMed, Web of Sciences, and Google Scholar to collect published data regarding *Rickettsia aeschlimannii* in different tick species, wild and domestic animals, humans, environment, and vegetation. The search was conducted by using some keywords, such as ticks, tick-borne pathogens, domestic animals, small ruminants, zoonosis, livestock, and *Rickettsia aeschlimannii*. Complete research articles, short communication, review papers, and conference articles were downloaded by using a combination of the above mentioned keywords. Lists of references from downloaded studies were examined to relevant articles (Table 2).

**Table 2:** Global epidemiology of *Rickettsia aeschlimannii* detected in different ticks and vertebrate hosts including human.

Continents	Country	Tick specie/Source	Host	Reference
Africa	Algeria	*Hy. scupense*	Sheep	Bitam et al. (2006)
		*Hy. marginatum marginatum*	Cattle, sheep	Bitam et al. (2006)	
		*Hy. aegyptium*	Tortoises	Bitam et al. (2009)	
		*Hy. marginatum rufipes*	Camels	Djerbouh et al. (2012)	
		*Hy. scupense*, *Hy. anatolicum*	Cattle	Leulmi et al. (2016)	
		*Hy. marginatum, Hy. scupense*, *Hy. impeltatum*	Cattle, goats, sheep, and equids	Sadeddine et al. (2020)	
	Angola	*Hy. turncatum*	Cattle	Palomar et al. (2022)	
	Benin	*A. variegatum*, *Rh. microplus*, *Hy. rufipes*	Cattle	Yessinou et al. (2023)	
	Cameroon	*Hy. rufipes, Hy. truncatum*	Cattle	Vanegas et al. (2018)	
		*Hy. rufipes, Hy. turncatum*	Nile Monitor, Hedgehog	Paguem et al. (2023)	
	Chad	*Hy. marginatum rufipes*	Camels	Mura et al. (2008b)	
	Egypt	*Hy. dromedarii*	Camels	Loftis et al. (2006)	
		*Hy. marginatum rufipes*	Cows		
		*Hy. impeltatum*	Cows		
		*Hy. anatolicum excavatum*, *Hy. impeltatum*, *Hy. dromedarii*, *Hy. marginatum marginatum*	Ruminants, camels, sheep, and cattle	Allam et al. (2018)	
	Ethiopia	*Hy. marginatum rufipes*	Cattle	Mura et al. (2008a)	
	Ghana	*A. variegatum*, *Hy. rufipes, Rh. evertsi, Rhipicephalus* sp.	Cattle, goats, and sheep	Addo et al. (2023)	
	Kenya	*Hy. truncatum, Hy. marginatum, Rh. pulchellus, A. variegatum, Hy. truncatum, Hyalomma* spp.	Camel, cattle, and goat	Koka et al. (2017)	
		*Hy. truncatum, Hy. marginatum rufipes, Rh. pulchellus*	Sheep and goats	Omondi et al. (2017)	
		*Hy. truncatum*	Domestic animals	Mutai et al. (2013)	
		*Rh. sanguineus, Hy. truncatum, Hy. marginatum rufipes*	Domestic animals	Diarra et al. (2017)	
		*Rh. pulchellus, Hy. dromedarii, Hy. rufipes, Hy. truncatum, Hy. impeltatum*	Camels and sheep	Getange et al. (2021)	
		*A. gemma*	Cattle	Godani et al. (2022)	
	Mali	*Hy. marginatum rufipes*	Cattle	Parola et al. (2001)	
	Morocco	Unknown	Human	Raoult et al. (2002)	
		*Hy. marginatum*	Cattle	Beati et al. (1997)	
	Niger	*Hy. marginatum rufipes*	Cattle	Parola et al. (2001)	
	Nigeria	*Hy. impeltatum, Hy. rufipes*	Camels	Kamani et al. (2015)	
		*Hy. dromedarii, Hy. truncatum, Hy. rufipes, Hy. impeltatum*	Camels	Onyiche et al. (2020)	
	Senegal	*Hy. marginatum rufipes, Hy. truncatum, Rh. evertsi evertsi*	Cows, donkeys, sheep, goats, and horses	Mediannikov et al. (2010)	
		*Hy. m. rufipes, Rh. evertsi, Hy. impeltatum*	Domestic animals	Sambou et al. (2014)	
	South Africa	*Rh. appendiculatus*	Human	Pretorius and Birtles (2002)	
		*Hy. marginatum*	Domestic animals	Sarih et al. (2008)	
		*Rh. appendiculatus*	Cattle	Pretorius and Birtles (2002)	
		*Hy. rufipes, Rh. appendiculatus, Rh. evertsi*	Donkeys	Halajian et al. (2018)	
	Sudan	*Hy. dromedarii, Hy. truncatum*	Camels	Morita et al. (2004)	
		*Rh. evertsi, Hy. rufipes*	Domestic animals	Springer et al. (2020)	
		*Hy. rufipes, Hy. dromedarii*	Camels	Shuaib et al. (2020)	
	Tunisia	*Hy. dromedarii*	Camel	Demoncheaux et al. (2012)	
		Unknown	Human	Znazen et al. (2006)	
		*Rh. sanguineus*	Dogs	Khrouf et al. (2014)	
		*Hy. impeltatum, Hy. dromedarii*	Camels	Selmi et al. (2020)	
		*Hy. marginatum, Rh. sanguineus*	Cattle	Kratou et al. (2023)	
	Zambia	*Hy. truncatum*	Dogs and cattle	Qiu et al. (2022)	
	*Hyalomma* spp.	Cattle	Chitanga et al. (2021)
Asia				
		*H. punctata*	Cattle, sheep	Song et al. (2018)
		*Rh. turanicus*	Sheep	Wei et al. (2015)
		*Hy. marginatum*	Yaks	Jiao et al. (2022)
	Iran	*Rh. sanguineus, Hy. rufipes,*	Unknown	Saman and Chegeni (2022)
		*Hy. marginatum, Rh. sanguineus*	Dogs, sheep	Ghasemi et al. (2022)
	Israel	*Hy. turanicum, Hy. dromedarii, Hy. excavatum*	Camel	Kleinerman et al. (2013)
		*Rh. sanguineus*	Dogs	Mumcuoglu et al. (2022)
		*Hy. marginatum*	Horse	Azagi et al. (2017)
	Kazakhstan	*H. punctata*	Vegetation	Shpynov et al. (2004)
	Lebanon	*Rh. annulatus, Hy. anatolicum, Rh. sanguineus*	Farms	De Mera et al. (2018)
Europe	Austria	*Hy. marginatum*	Migratory birds	Duscher et al. (2018)
	Bulgaria	*Hy. anatolicum, Hy. marginatum, Hy. excavatum, Rhipicephalus* spp.	Dogs and cattle	Nader et al. (2018)
	Croatia	*Hy. marginatum*	Cattle	Punda-Polic et al. (2003)
	England	*Hy. marginatum*	Human	McGinley et al. (2021)
	France	*Hy. marginatum*	Sheep, humans, cattle, vegetation, wild boar, cattle, sheep, humans (on clothes), and vegetation	Matsumoto et al. (2004)
		*Hyalomma* sp.	Migratory birds	Socolovschi et al. (2011)
		*Hy. marginatum rufipes*	Migratory birds	Matsumoto et al. (2004)
		*Hy. marginatum, Rh. sanguineus, Hy. scupense, Rh. bursa*	Domestic and wild animals	Grech-Angelini et al. (2020)
	Germany	*Hy. marginatum*	Migratory birds	Rumer et al. (2011)
		*Hy. marginatum, Hy. rufipes*	Sheep, horse, and cat	Chitimia-Dobler et al. (2019)
	Georgia	*H. sulcata, Hy. scupense, Hy. marginatum, H. punctata,*	Cows and dogs	Sukhiashvili et al. (2020)
	Greece	*Hy. aegyptium*	Wild birds	Diakou et al. (2016)
		*Hy. anatolicum*	Sheep	Psaroulaki et al. (2006)
	Greece and Italy	*Hy. marginatum, Hy. rufipes, I. frontalis, Haemaphysalis* sp.	Migratory birds	Wallmenius et al. (2014)
	Serbia	*I. ricinus*	Humans	Banović et al. (2022)
	Italy	*Hy. lusitanicum*	Humans	Otranto et al. (2014)
		*Hy. lusitanicum, Hy. marginatum, D. marginatus, I. ricinus*	Humans	Blanda et al. (2017)
		*I. ricinus, Rh. turanicus*	Insugherata Natural Reserve	Mancini et al. (2015)
		*Hy. marginatum, Hy. lusitanicum*	Humans, domestic mammals, and wildlife	Chisu et al. (2018)
		*Hy. marginatum*	Herbivores (donkeys, cattle, and sheep)	Beninati et al. (2005)
		*D. marginatus*	Dogs	Sgroi et al. (2022)
		*Hy. marginatum*	Vegetation	Tomassone et al. (2013)
		*Hy. marginatum, Hy. truncatum, Hy. rufipes, Hyalomma* sp.	Great reed warbler, Reed warbler, Icterine warbler, Western yellow wagtail, Whinchat, Garden warbler, European pied flycatcher, Sedge warbler, Spotted flycatcher, Common whitethroat, Common redstart, Common nightingale, Collared flycatcher, Subalpine warbler, and Northern wheatear	Pascucci et al. (2019a,b)
		*Hy. rufipes, A. marmoreum, Hyalomma* sp.	Black redstart, Pied flycatcher, Redstart, Tree pipit, Whinchat, Whitethroat, Collared flycatcher, European robin, Northern weathear, Redstart, Song thrush, Whinchat, Whitethroat, and Wood warbler	Battisti et al. (2020)
		*Hy. rufipes*	Whitethroat tree pipit, Northern, Wheatear, and Whinchat	Rollins et al. (2021)
		Human	Human	Tosoni et al. (2016)
		*Hy. marginatum*	Wild boar	Maioli et al. (2009)
		*Hy. m. marginatum, Hyalomma* spp.*, Amblyomma* sp.	Migratory birds	Toma et al. (2014)
		*Hy. truncatum, Hy. rufipes*	Cattle	Tomassone et al. (2016)
		*Hy. marginatum rufipes*	Cattle	Ehounoud et al. (2016)
		*Hy. marginatum*	Free living	Scarpulla et al. (2016)
		*Hy. m. marginatum*	Mouflon	Chisu et al. (2018)
		*Hy. marginatum*	*C. elaphus*	Pascucci et al. (2019a,b)
	Poland	*D. reticulatus*	Human	Koczwarska et al. (2023)
	Portugal	*Hy. marginatum*	Wild birds	Santos-Silva et al. (2006)
	Russia (western Russia)	*Hy. marginatum*	Cattle and vegetation	Shpynov et al. (2009)
	Romania	*Hy. marginatum*	Cattle	Andersson et al. (2018)
		*H. concinna*	Hedgehogs	Borşan et al. (2021)
	Spain	*H. inermis*	Vegetation	Portillo et al. (2008)
		*Hy. marginatum*	Humans	Fernández-Soto et al. (2009)
		*Hy. marginatum, H. punctata, I. ricinus, Rh. bursa, Rh. turanicus, Rh. sanguineus*	Humans	Fernández-Soto et al. (2003)
		*Hy. marginatum*	Humans and cows	Oteo et al. (2005)
	Spain and Morocco	*Hy. marginatum*	Humans and birds	Palomar et al. (2016)
	Sweden	*Hy. rufipes, Hy. marginatum*	Humans, Horse	Grandi et al. (2020)
	Turkey	*Hy. marginatum*	Humans	Keskin et al. (2016)
		*Hy. marginatum*	Humans	Keskin and Bursali, 2016
		*Rh. bursa, Hy. marginatum, Hyalomma* sp.	Humans	Gargili et al. (2012)
		*Hy. marginatum*	Humans	Bursalı et al. (2017)
		*Hy. marginatum, Hy. aegyptium, Rh. turanicus, H. parva, H. punctata, H. sulcata, D. marginatus, I. ricinus, Hyalomma* spp.	Humans	Karasartova et al. (2018)
		*Hy. scupense, Hy. aegyptium, Hy. marginatum, Hy. excavatum*	Humans	Orkun et al. (2014)
		*Rh. turanicus, Hy. marginatum*	Wild boar	Orkun and Çakmak (2019)
		*Hy. aegyptium*	Hedgehogs	Orkun and Çakmak (2019)
		*Hy. marginatum, Rh. bursa*	Cattle, goat	Demir et al. (2020)
		*Hy. aegyptium*	*Testudo graeca*	Akveran et al. (2020)
	United Kingdom	*Hy. rufipes*	Horse	Hansford et al. (2019)
South America	Bolivia	*A. tigrinum*	Dogs	Tomassone et al. (2010)
North America	Panama	*A. dissimile*	*Boa constrictor, Dasypus novemcinctus*	Bermudez et al. (2021)

## Results

### Identified ticks

A total of 369 domestic animals were examined in 7 districts including Mohmand (23 cattle, 14 goats, and 12 sheep), Dir Upper (20 cattle, 15 goats, 12 sheep, and 7 dogs), Dir lower (26 cattle, 17 goats, 13 sheep, and 5 dogs), Bajaur (19 cattle, 16 goats, 13 sheep, and 11 dogs), Charsadda (17 cattle, 15 goats, 13 sheep, and 5 dogs), Mardan (16 cattle, 13 goats, 20 sheep, and 5 dogs), and Swabi (17 cattle, 13 goats, 10 sheep, and 4 dogs) (*p* = 0.248). Of the examined hosts, 66% (247 of 369) of hosts were parasitized by 872 ticks of various life stages (Table 3), having a mean intensity of 3.5 ticks/infested host while the mean abundance was 2.3 ticks/examined host. The highest tick infestation was found on goats (74 of 103, 71.8%) compared with cattle (99 of 138, 71.7%), sheep (62 of 91, 60%), and dogs (12 of 37, 32.4%) (*p* < 0.0001). All collected ticks belonged to three different genera of ixodid ticks, namely, *Rhipicephalus, Hyalomma,* and *Haemaphysalis.* Adult female including engorged ticks were the most prevalent (376, 43.1%), followed by nymphs (303, 34.74%) and males (193, 22.13%) (Table 3) (*p* < 0.0001). The highest tick burden was observed on domestic animals in the Bajaur district (19.3%), followed by Mardan (15%), Swabi (14.9%), Charsadda (13.9%), Mohmand (13.3%), Dir Upper (12.7%), and Dir Lower (10.6%) (*p* < 0.0001). The most dominant species was *Rhipicephalus microplus* (19.0%), followed by *Hyalomma anatolicum* (15.7%), *Rhipicephalus turanicus* (11.5%), *Haemaphysalis sulcata* (10.6%), *Haemaphysalis bispinosa* (9.8%), *Haemaphysalis montgomeryi* (9.1%), *Rhipicephalus sanguineus* (8.8%), *Hyalomma scupense* (4.9%), *Rhipicephalus haemaphysaloides* (3.8%), *Haemaphysalis cornupunctata* (2.7%) *Hyalomma isaaci* (1.9%), and *Hyalomma turanicum* (1.60%) (*p* < 0.0001).

**Table 3:** Occurrence of ticks infesting various domestic animals and detection of rickettsial DNA associated with ticks.

District/Areas	Host	No. of examined host	No. of infested host	Collected Tick species	No. of Nymphs/Female/male	Total No. of ticks	Molecularly screened Ticks*	*Rickettsia* spp. detected via *gltA, ompA, ompB*
Mohmand/ Rural	Cattle	23	15	*Hy. anatolicum*	8 N/3F/2F*/2M	15	3N,1F	0
				*Rh. microplus*	8 N/7F/3F*/7M	25	3N,1F	0
	Goats	14	9	*Hy. turanicum*	5N/5F/1F*/3M	14	2N,2F	2N,1F
				*H. sulcata*	6N/7F/3M	16	2N,1F,1M	0
	Sheep	12	8	*Hy. isaaci*	6N/7F/4M	17	2N,2F	0
				*H. bispinosa*	6N/7F/3M	16	2N,1F,1M	1N,1F
				*H. montgomeryi*	3N/8F/2M	13	2N,2F	0
Total		49	32 (8.6%)		42N/44F/6F*/24M	116 (13.3%)	16N,10F,2M	3N,2F
Dir Upper/ Rural	Cattle	20	14	*Rh. haemaphysaloides*	2N/6F/2M	10	2N,2F	0
				*Rh. microplus*	9N/7F/3F*/8M	27	2N,2F	0
	Goats	15	9	*Rh. turanicus*	4N/5F/2M	11	2N,2F	0
				*H. montgomeryi*	2N/8F/2M	12	2N,2F	0
	Sheep	10	8	*H. sulcata*	5N/6F/2M	13	2N,2F	0
				*Hy. scupense*	2N/6F/1M	9	2N,2F	0
				*Hy. anatolicum*	5N/3F/3F*/4M	15	2N,2F	0
	Dogs	7	3	*Rh. sanguineus*	5N/6F/3M	14	2N,2F	0
Total		52	34 (9.2%)		34N/47F/6F*/24M	111 (12.7%)	16N,16F	0
Dir lower/Rural	Cattle	26	18	*Rh. microplus*	10N/6F/4F*/9M	29	2N,2F,1M	0
	Goats	17	11	*H. bispinosa*	2N/8F/2M	12	3N,2F	0
				*H. montgomeryi*	2N/10F/3M	15	2N,2F,1M	IN,1F
	Sheep	13	7	*H. sulcata*	2N/8F/3M	13	3N,2F	0
	Dogs	5	2	*Rh. turanicus*	4N/5F/3M	12	3N,2F	0
				*Rh. sanguineus*	5N/3F/2F*/2M	12	3N,2F	0
Total		61	38 (10.2%)		25N/40F/6F*/22M	93 (10.6%)	16N,12F,2M	1N,1F
Bajaur/Rural	Cattle	19	17	*Rh. microplus*	10N/8F/2F*/8M	28	2N,1F	0
				*Hy. anatolicum*	6N/5F/3F*/7M	21	2N,1F	0
	Goats	16	14	*H. sulcata*	7N/8F/4M	19	2N,1F	0
				*H. montgomeryi*	7N/8F/3M	18	2N,1F	0
				*Rh. turanicus*	5N/4F/2M	11	2N,1F	1N
	Sheep	13	10	*Rh. turanicus*	8N/4F/2F*/3M	17	1N,1F,1M	0
				*H. sulcata*	7N/8F/3M	18	1N,1F,1M	0
				*H. bispinosa*	8N/9F/5M	22	2N,1F	0
	Dogs	11	2	*Rh. sanguineus*	5N/5F/2F*/3M	15	1N,1F,1M	0
Total		59	43 (11.65%)		63N/59F/9F*/38M	169 (19.3%)	15N,9F,3M	1N
Charsadda/Urban	Cattle	17	9	*Rh. microplus*	8N/4F/4F*/7M	23	2N,2F	0
				*Rh. haemaphysaloides*	6N/4F/2M	12	3N,1F	0
	Goats	15	12	*Rh. haemaphysaloides*	3N/8F/1M	12	3N,1F	0
				*Rh. turanicus*	3N/4F/2M	9	2N,2F	0
				*Hy. anatolicum*	6N/4F/2F*/4M	16	2N,1F,1M	0
	Sheep	13	8	*H. cornupunctata*	2N/5F/1M	8	2N,2F	0
				*H. bispinosa*	2N/4F/1M	7	2N,2F	0
				*Hy. anatolicum*	5N/3F/3F*/3M	14	2N,2F	0
	Dogs	5	2	*Rh. turanicus*	4N/5F/2M	11	2N,2F	0
				*Rh. sanguineus*	4N/4F/2M	10	3N,1F	0
Total		50	31 (8.40%)		43N/45F/9F*/25M	122 (13.9%)	23N,16F,1M	0
Mardan/Urban	Cattle	16	13	*Rh. microplus*	6N/4F/2F*/4M	16	2N,1F	0
				*Hy. anatolicum*	5N/3F/2F*/4M	14	2N,1F	0
	Goats	13	9	*Rh. turanicus*	4N/3F/2M	10	2N,1F	0
				*Hy. anatolicum*	4N/5F/2F*/2M	11	2N,1F	0
	Sheep	20	14	*H. cornupunctata*	6N/6F/4M	16	2N,1F	0
				*Hy. scupense*	5N/8F/3M	18	2N,1F	0
				*H. bispinosa*	5N/5F/2F*/3M	15	1N,1F,1M	0
				*H. montgomeryi*	2N/4F/2M	6	1N,1F,1M	0
	Dogs	5	2	*Rh. sanguineus*	5N/6F/3M	14	2N,1F	0
				*Rh. turanicus*	4N/4F/3M	11	1N,1F,1M	0
Total		54	38 (10.2%)		47N/46F/8F*/30M	131 (15.0%)	17N,10F,3M	0
Swabi/Urban	Cattle	17	13	*Rh. microplus*	6N/5F/3F*/4M	18	3N,1F	0
				*Hy. anatolicum*	5N/4F/2F*/3M	14	2N,1F,1M	0
				*Rh. turanicus*	4N/3F/2M	9	2N,1F,1M	0
	Goats	13	10	*H. bispinosa*	5N/5F/4M	14	2N,1F,1M	0
				*H. montgomeryi*	6N/6F/4M	16	2N,2F	0
	Sheep	10	7	*Hy. scupense*	6N/4F/2F*/4M	16	2N,1F,1M	0
				*Hy. anatolicum*	7N/4F/2F*/4M	17	2N,2F	0
				*H. sulcata*	5N/6F/3M	14	2N,2F	0
	Dogs	4	1	*Rh. sanguineus*	5N/5F/2M	12	2N,2F	0
Total		44	31 (8.40%)		49N/42F/9F*/30M	130 (14.90)	19N,13F,4M	0
Overall total		369	(66.65)		303N/323F/53F*/193M	872	122N,86F,15M	5N,3F

N, nymphs; F, Adult females; F*, Engorged females; M, males. *Ticks subjected to PCR to screen for *Rickettsia* spp.

### Detection of *Rickettsia* spp. in ticks

DNA of a subset of 223 ticks was used for the detection of *Rickettsia* spp. by the amplification of three rickettsial markers, namely, *gltA*, *ompA*, and *ompB* gene fragments. Positive ticks for rickettsial *gltA* were also positive when tested with the primers of *ompA* and *ompB*. In total, 8 out of 223 (3.58%) ticks including 5 of 28 (17.85%) from Mohmand, 2 of 30 (6.6%) from Dir Lower, and 1 of 27 (3.7%) from Bajaur were found positive for *Rickettsia* spp. (*P*, 0.1621). Rickettsial DNA was found in three tick species, namely, *H. montgomeryi*, *H. bispinosa*, and *H. turanicum,* which was collected from sheep and goats. The overall prevalence of *Rickettsia* spp. was 3.58% (8 of 223). No rickettsial DNA was detected in *Rh. microplus* (27)*, Hy. isaaci* (4)*, Hy. scupense* (11)*, Rh. turanicus* (33)*, Hy. anatolicum* (33)*, Rh. haemaphysaloides* (12)*, Rh. sanguineus* (23)*, H. cornupunctata* (7), and *H. sulcata* (23). Information regarding the prevalence of *Rickettsia* spp. in various ticks is shown in Table 3.

### Sequence and phylogenetic analysis

Assembled contigs of direct and reverse sequence reads for each PCR-amplified fragment were analyzed. Due to a single haplotype, consensus sequences were generated for each partial gene. The consensus sequence of *gltA* (348 bp) showed 100% identity with *R. aeschlimannii* from Russia, Senegal, and Kazakhstan, followed by 99.71% identity with *R. aeschlimannii-*type strain from Morocco. Similarly, 100% identity was shown by the obtained *ompA* (467 bp) consensus sequence with *R. aeschlimannii* from Russia, Spain, Turkey, and Kazakhstan, followed by 99.79% identity with *R. aeschlimannii-*type strain. Similarly, the *ompB* (764 bp) consensus sequence also showed 100% identity with *R. aeschlimannii* from Kazakhstan, Russia, Italy, and Portugal, followed by 99.74% identity with *R. aeschlimannii-*type strain. In all cases, the query coverage was 100%. The obtained rickettsial *gltA* sequence (accession number: OR351959), *ompA* sequence (accession number: OR351960), and *ompB* sequence (accession number: OR351961) were submitted to GenBank.

**Figure 2:**
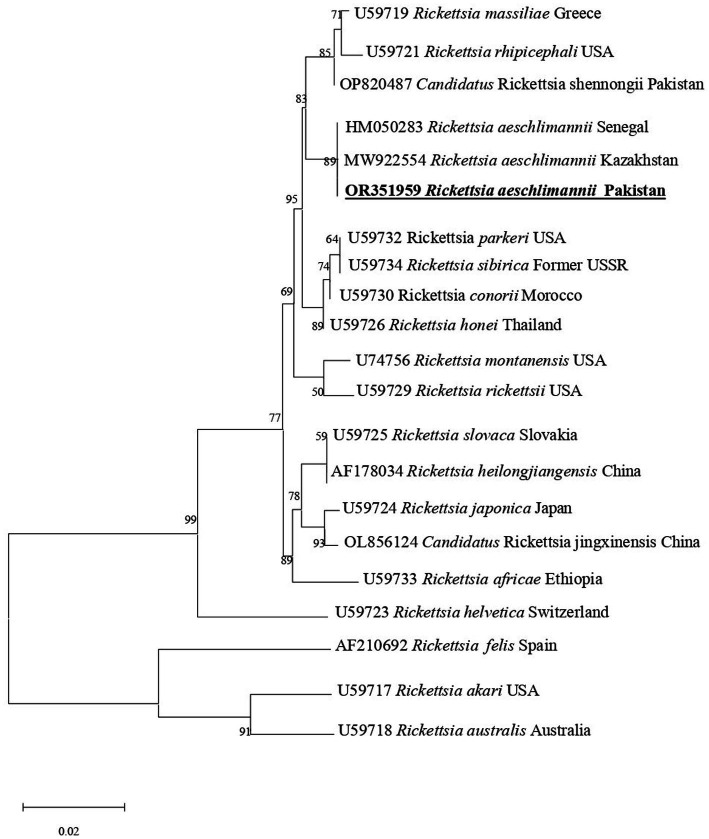
Phylogenetic analysis based on the *gltA* sequences of *Rickettsia aeschlimannii.* The obtained sequences of the present study are indicated in bold and underlined fonts. *Rickettsia akari* and *Rickettsia australis* were used as out-group.

**Figure 3:**
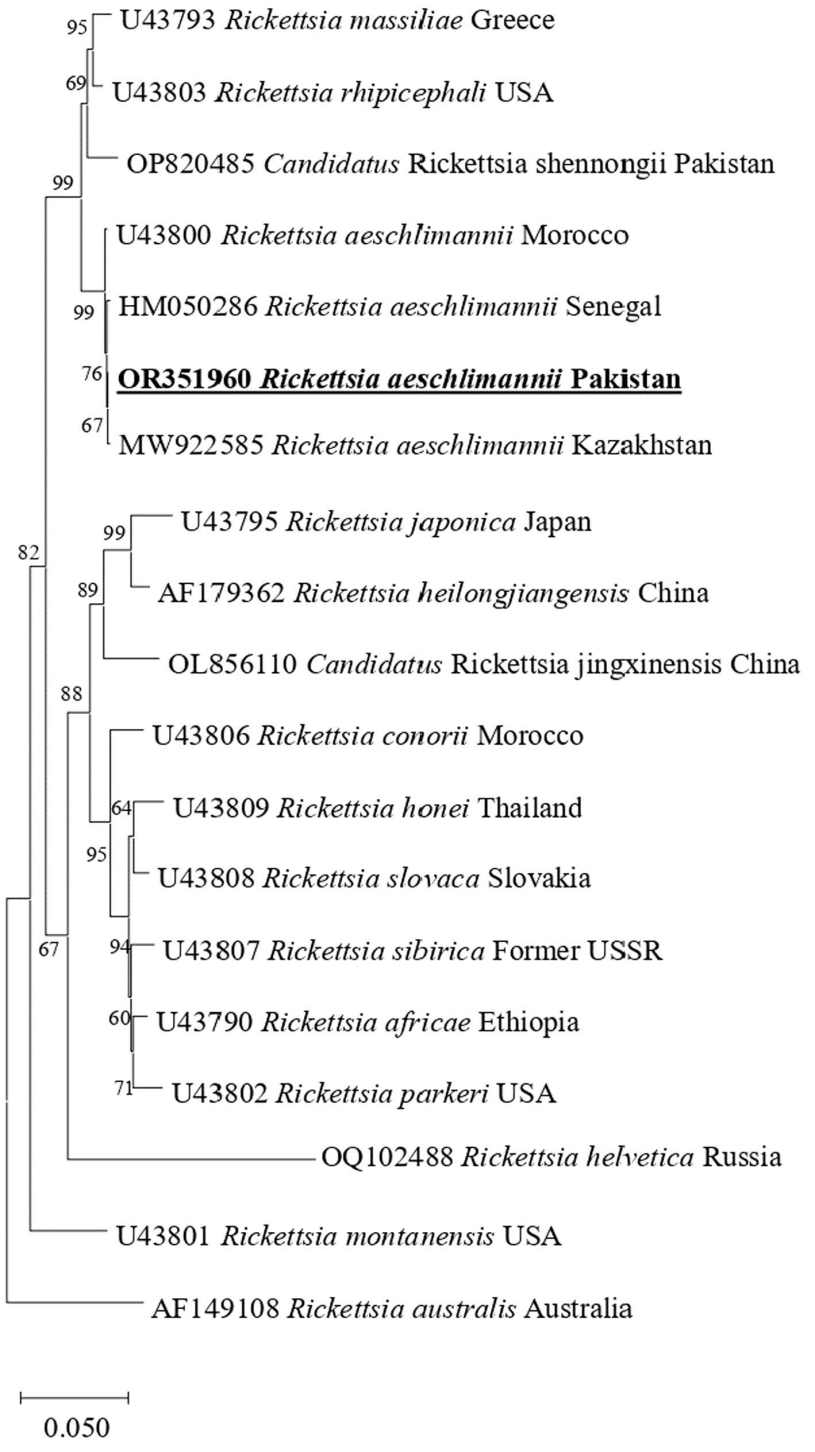
Phylogenetic analysis based on the *ompA* sequences of *R. aeschlimannii.* The sequences obtained in this study are indicated in bold and underlined fonts. *Rickettsia australis* is used as an out-group.

**Figure 4:**
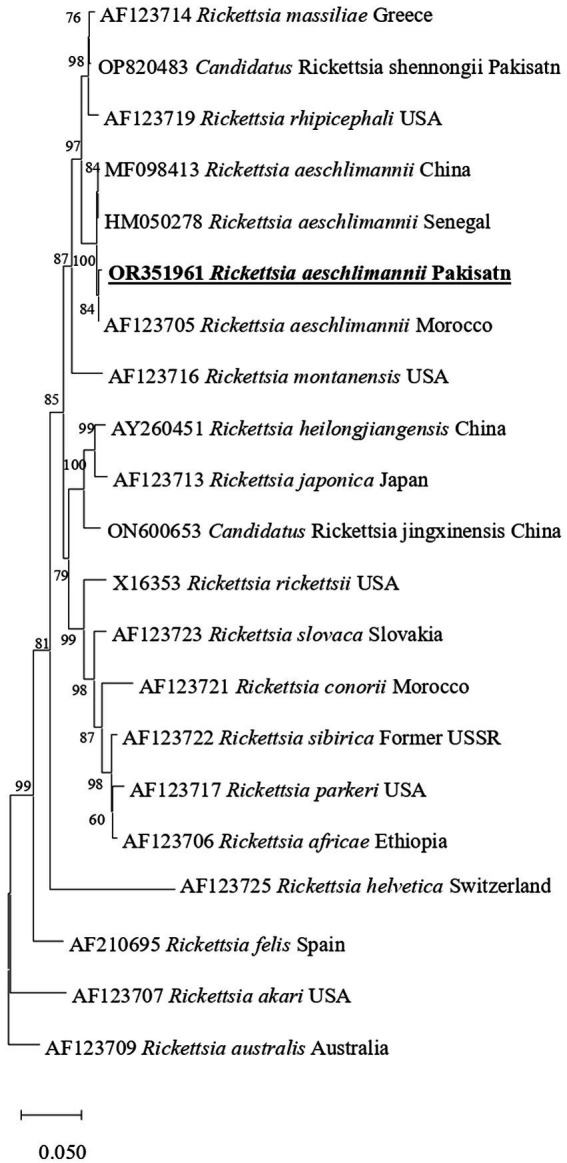
Phylogenetic analysis based on the sequences of the *ompB* genes of *R. aeschlimannii.* The obtained sequences in the present study are indicated in bold and underlined fonts. *Rickettsia australis* used as an out-group.

In phylogenetic tree based on rickettsial *gltA*, *R. aeschlimannii* clustered with *R. aeschlimannii* reported from Senegal (HM050283) and Kazakhstan (MW922554) (Figure 2). Rickettsial *ompA* clustered with *R. aeschlimannii* reported from Senegal (HM050286), Kazakhstan (MW922585), and Morocco (U43800) (Figure 3), while *ompB* sequence clustered with *R. aeschlimannii* reported from China (MF098413), Senegal (HM050278), and Morocco (AF123705) (Figure 4).

## Discussion

Previous studies have recorded the presence of *Rickettsia* spp. in different tick-infesting hosts in Pakistan. However, there was a paucity of information regarding *R. aeschlimannii* in the region. This study presents the first report on molecular detection of *R. aeschlimannii* in *H. bispinosa*, *H. montgomeryi,* and *Hy. turanicum* ticks collected from sheep and goats in Pakistan. The obtained sequences showed maximum identity and phylogenetically clustered with *Rickettsia aeschlimannii,* which confirms the occurrence of *Rickettsia aeschlimannii* in the regions. *Rickettsia aeschlimannii*, an emerging pathogen with zoonotic potential, has been observed to cause infections in humans across various countries such as Morocco (Raoult et al., 2002), Tunisia (Znazen et al., 2006), Algeria (Mokrani et al., 2008), Greece (Germanakis et al., 2013), and Russia (Igolkina et al., 2022). The detection of *R. aeschlimannii* in tick-parasitizing domestic animals suggests a high exposure of livestock holders to this pathogen.

Small ruminants (goats and sheep), domestic dogs, and cattle were found infested by the ticks of genera *Rhipicephalus, Hyalomma,* and *Haemaphysalis*. Among the collected ticks, *Rh*. *microplus*, *Hy*. *anatolicum*, *H*. *bispinosa* and *H*. *montgomeryi* were the dominant tick species. These findings mirror the pattern observed in other previous studies conducted in same region (Karim et al., 2017; Ali et al., 2019; Alam et al., 2022; Khan Z. et al., 2022; Tila et al., 2023) underscoring the regional significance of these tick species. Furthermore, it emphasizes the need for further research to comprehensively investigate their prevalence, distribution, and potential implications for public health (Ali et al., 2023).

Ticks of different genera have been reported as carriers for various *Rickettsia* spp. in Pakistan (Ali et al., 2021, 2022; Khan M. et al., 2022; Khan Z. et al., 2022; Numan et al., 2022; Ullah et al., 2023). Herein, *Rickettsia aeschlimannii* was detected in *H. bispinosa*, *H. montgomeryi,* and *Hy. turanicum* ticks using three genetic markers, namely, *gltA*, *ompA*, and *ompB*. To date, there has been a lack of information regarding the detection of *R. aeschlimannii* in *H. bispinosa* and *H. montgomeryi* tick-infesting domestic animals, such as goats and sheep. There is also a possibility that the rickettsial DNA is detected in the ticks may be due to ingesting rickettsemic host blood. Literature search revealed that *R. aeschlimannii* is associated with a variety of tick species belonging to six genera of hard ticks, namely, *Hyalomma*, *Rhipicephalus, Haemaphysalis, Amblyomma, Dermacentor*, and *Ixodes* (Parola et al., 2001; Fernández-Soto et al., 2003; Shpynov et al., 2004; Tomassone et al., 2010; Karasartova et al., 2018). Furthermore, there are limited reports, which have detected this pathogen in Asia (Wei et al., 2015; Satjanadumrong et al., 2019). This study found its association with two new ticks, expanding its known host and geographical range. The detection of *R. aeschlimannii* in *Hyalomma* and *Haemaphysalis* ticks suggests a potential threat to livestock holders. Additionally, the rate of *R. aeschlimannii* was observed highest in *Haemaphysalis* ticks, which are the primary ticks that infest goats and sheep. This enhances the feasibility of public health risks as these ticks may occasionally infest humans (Guglielmone and Robbins, 2018). *Rickettsia aeschlimannii* has been detected in all life stages of ticks, such as adult females, males, larvae, and nymphs (Raoult et al., 2002; Shpynov et al., 2009; Germanakis et al., 2013; Orkun et al., 2014; Wallmenius et al., 2014; Tosoni et al., 2016). This study presents the first molecular evidence of *R. aeschlimannii* in *H. bispinosa* and *H. montgomeryi* ticks, which suggests that other tick species could also serve as competent vectors for this pathogen in the region.

Molecular methods are considered faster and more accurate for the genetic characterization and phylogenetic analysis of *Rickettsia* spp. (Fournier et al., 1998; Roux and Raoult, 2000). The *gltA*, *ompA, ompB,* and *sca*4 DNA sequences have been used as suitable genetic markers to discriminate different *Rickettsia* spp (Roux and Raoult, 2000; Fournier et al., 2003; Labruna et al., 2004). Herein, these sequences were targeted for molecular characterization and phylogenetic analysis of *R. aeschlimannii* which revealed the close relatedness with corresponding species of the SFG. We assume that human infection caused by rickettsial agents of SFG maybe underreported due to the lack of epidemiological information among health practitioners and laboratory technicians and the lack of diagnostic procedures in Pakistan, given the relevance of the occurrence of these agents in the region. Further studies in the region should be encouraged to obtain information on zoonotic outcomes due to these infectious agents.

## Conclusion

This study for the first time contributes to the neglected knowledge and genetic characterization of tick-borne *R. aeschlimannii* in *H. bispinosa*, *H. montgomeryi,* and *Hy. turanicum* ticks in Pakistan. The results of this study also indicated that goats and sheep are exposed to *R. aeschlimannii*. Further molecular studies are important to screen *R. aeschlimannii* in livestock and livestock holders who have close contact with domestic animals.

## Data availability statement

We have released your GenBank submissions in OR351959-OR351961. Your sequences will be available for public access within a few days. If you have additional information about your sequences or wish to make further revisions, see: https://www.ncbi.nlm.nih.gov/Genbank/update.html for proper update formats.

## Ethics statement

The design of the current work has received an approval from the Advanced Study and Research Board members (Dir/A&R/AWKUM/2023/0014) and the Faculty of Zoology department, Abdul Wali Khan University Mardan, Pakistan. Permissions were obtained from the animal’s owner before collecting ticks from their animals. The studies were conducted in accordance with the local legislation and institutional requirements. Written informed consent was obtained from the owners for the participation of their animals in this study.

## Author contributions

AM: Data curation, Investigation, Methodology, Software, Writing – original draft. MA: Data curation, Formal analysis, Funding acquisition, Investigation, Methodology, Writing – original draft, Writing – review & editing. AbdA: Funding acquisition, Investigation, Methodology, Project administration, Resources, Writing – original draft, Writing – review & editing. TT: Data curation, Formal analysis, Investigation, Writing – original draft, Writing – review & editing. T-YY: Formal analysis, Validation, Visualization, Writing – original draft, Writing – review & editing. K-HT: Data curation, Formal analysis, Funding acquisition, Investigation, Writing – original draft, Writing – review & editing. AbiA: Conceptualization, Funding acquisition, Investigation, Methodology, Project administration, Resources, Supervision, Writing –original draft, Writing – review & editing.

## References

[ref1] Addo S. O. Bentil R. E. Baako B. O. A. Yartey K. N. Behene E. Asiamah B.. (2023). Occurrence of *Rickettsia* spp. and *Coxiella burnetii* in ixodid ticks in Kassena-Nankana, Ghana. Exp. Appl. Acarol. 90, 137–153. doi: 10.1007/s10493-023-00808-0, PMID: 37322233

[ref2] Akveran G. A. Karasartova D. Keskin A. Comba A. Celebi B. Mumcuoglu K. Y.. (2020). Bacterial and protozoan agents found in *Hyalomma aegyptium* (L., 1758) (Ixodida: Ixodidae) collected from *Testudo graeca* L., 1758 (Reptilia: Testudines) in Corum Province of Turkey. Ticks Tick-borne Dis. 11:101458. doi: 10.1016/j.ttbdis.2020.101458, PMID: 32389537

[ref3] Alam S. Khan M. Alouffi A. Almutairi M. M. Ullah S. Numan M.. (2022). Spatio-temporal patterns of ticks and molecular survey of *Anaplasma marginale*, with notes on their phylogeny. Microorganisms 10:1663. doi: 10.3390/microorganisms10081663, PMID: 36014081 PMC9413622

[ref9012] Ali A. Khan M.A. Zahid H. Yaseen P. M. Qayash Khan M. Nawab J.. (2019). Seasonal dynamics, record of ticks infesting humans, wild and domestic animals and molecular phylogeny of *Rhipicephalus microplus* in Khyber Pakhtunkhwa Pakistan. Frontiers in Physiology 10:793. doi: 10.3389/fphys.2019.00793 31379587 PMC6646419

[ref4] Ali A. Ullah S. Numan M. Almutairi M. M. Alouffi A. Tanaka T. (2023). First report on tick-borne pathogens detected in ticks infesting stray dogs near butcher shops. Front. Vet. Sci. 10:1246871. doi: 10.3389/fvets.2023.1246871, PMID: 37799410 PMC10548827

[ref5] Ali A. Numan M. Khan M. Aiman O. Muñoz-Leal S. Chitimia-Dobler L.. (2022). *Ornithodoros (Pavlovskyella)* ticks associated with a *Rickettsia* sp. in Pakistan. Parasit. Vect. 15, 1–13. doi: 10.1186/s13071-022-05248-0, PMID: 35449077 PMC9026656

[ref6] Ali A. Zahid H. Zeb I. Tufail M. Khan S. Haroon M.. (2021). Risk factors associated with tick infestations on equids in Khyber Pakhtunkhwa, Pakistan, with notes on *Rickettsia massiliae* detection. Parasit. Vectors 14, 363–312. doi: 10.1186/s13071-021-04836-w, PMID: 34256806 PMC8276440

[ref8] Allam N. El Moghazy F. M. Abdel-Baky S. (2018). Molecular epidemiological updates on spotted fever rickettsioses in animal species and their hard ticks settling Egyptian desert. J. Adv. Pharm. Educat. Res. 8:65.

[ref9] Aneela A. Almutairi M. M. Alouffi A. Ahmed H. Tanaka T. da Silva Vaz I.. (2023). Molecular detection of *Rickettsia hoogstraalii* in *Hyalomma anatolicum* and *Haemaphysalis sulcata*: updated knowledge on the epidemiology of tick-borne *Rickettsia hoogstraalii*. Vet. Sci. 10:605. doi: 10.3390/vetsci10100605, PMID: 37888557 PMC10611279

[ref10] Andersson M. O. Tolf C. Tamba P. Stefanache M. Radbea G. Frangoulidis D.. (2018). Molecular survey of neglected bacterial pathogens reveals an abundant diversity of species and genotypes in ticks collected from animal hosts across Romania. Parasit. Vectors 11, 1–10. doi: 10.1186/s13071-018-2756-1 29554947 PMC5859542

[ref11] Apanaskevich D. A. Filippova N. A. Horak I. G. (2010). The genus *Hyalomma* Koch, 1844. X. Redescription of all parasitic stages of *Hy. scupense*, 1919 (*Hy. detritum* Schulze) (Acari: Ixodidae) and notes on its biology. Folia Parasitol. 57, 69–78. doi: 10.14411/fp.2010.009 20450001

[ref12] Apanaskevich D. A. Schuster A. L. Horak I. G. (2008). The genus *Hyalomma*: VII. Redescription of all parasitic stages of *Hy. dromedarii* and *Hy. schulzei* (Acari: Ixodidae). J. Med. Entomol. 45, 817–831. doi: 10.1093/jmedent/45.5.817 18826023

[ref13] Azagi T. Klement E. Perlman G. Lustig Y. Mumcuoglu K. Y. Apanaskevich D. A.. (2017). Francisella-like endosymbionts and *Rickettsia* species in local and imported *Hyalomma* ticks. Appl. Environ. Microbiol. 83, e01302–e01317. doi: 10.1128/AEM.01302-17, PMID: 28710265 PMC5583492

[ref14] Banović P. Díaz-Sánchez A. A. Simin V. Foucault-Simonin A. Galon C. Wu-Chuang A.. (2022). Clinical aspects and detection of emerging rickettsial pathogens: A “One Health” approach study in Serbia, 2020. Frontiers in microbiology 12, p.797399. doi: 10.3389/fmicb.2021.797399, PMID: 35154030 PMC8825779

[ref15] Battisti E. Urach K. Hodžić A. Fusani L. Hufnagl P. Felsberger G.. (2020). Zoonotic pathogens in ticks from migratory birds, Italy. Emerg. Infect. Dis. 26, 2986–2988. doi: 10.3201/eid2612.181686, PMID: 33219656 PMC7706978

[ref16] Beati L. Meskini M. Thiers B. Raoult D. (1997). *Rickettsia aeschlimannii* sp. nov., a new spotted fever group *Rickettsia* associated with *Hyalomma marginatum* ticks. Int. J. Syst. Evol. Microbiol. 47, 548–554. doi: 10.1099/00207713-47-2-548 9103647

[ref17] Beninati T. Genchi C. Torina A. Caracappa S. Bandi C. Lo N. (2005). Rickettsiae in ixodid ticks, Sicily. Emerg. Infect. Dis. 11, 509–511. doi: 10.3201/eid1103.040812, PMID: 15789496 PMC3298249

[ref18] Bermudez S. Martinez-Mandiche J. Dominguez L. Gonzalez C. Chavarria O. Moreno A.. (2021). Diversity of *Rickettsia* in ticks collected from wild animals in Panama. Ticks Tick-borne Dis. 12:101723. doi: 10.1016/j.ttbdis.2021.101723, PMID: 33857748

[ref19] Bitam I. Kernif T. Harrat Z. Parola P. Raoult D. (2009). First detection of *Rickettsia aeschlimannii* in *Hyalomma aegyptium* from Algeria. Clin. Microbiol. Infect. 15, 253–254. doi: 10.1111/j.1469-0691.2008.02274.x, PMID: 19548989

[ref20] Bitam I. Parola P. Matsumoto K. Rolain J. M. Baziz B. Boubidi S. C.. (2006). First molecular detection of *R. conorii*, *R. aeschlimannii*, and *R. massiliae* in ticks from Algeria. Ann. N. Y. Acad. Sci. 1078, 368–372. doi: 10.1196/annals.1374.073 17114743

[ref21] Blanda V. Torina A. La Russa F. D’Agostino R. Randazzo K. Scimeca S.. (2017). A retrospective study of the characterization of *Rickettsia* species in ticks collected from humans. Ticks Tick-borne Dis. 8, 610–614. doi: 10.1016/j.ttbdis.2017.04.005, PMID: 28457821

[ref22] Borşan S. D. Ionică A. M. Galon C. Toma-Naic A. Peştean C. Sándor A. D.. (2021). High diversity, prevalence, and co-infection rates of tick-borne pathogens in ticks and wildlife hosts in an urban area in Romania. Front. Microbiol. 12:645002. doi: 10.3389/fmicb.2021.645002, PMID: 33767683 PMC7985354

[ref23] Boulanger N. Boyer P. Talagrand-Reboul E. Hansmann Y. (2019). Tick-borne diseases. Medecine et Maladies Infectieuses 49, 87–97. doi: 10.13140/RG.2.2.18223.30881 30736991

[ref24] Brouqui P. Parola P. Fournier P. E. Raoult D. (2007). Spotted fever rickettsioses in Southern and Eastern Europe. FEMS 49, 2–12. doi: 10.1111/j.1574-695X.2006.00138.x, PMID: 17266709

[ref25] Bursalı A. Keskin A. Keskin A. Kul Köprülü T. Tekin Ş. (2017). Investigation of the presence of Rickettsiae in ticks parasitizing humans in Corum Region. Turk. Hij. Tecr. Biyol. Derg. 74, 293–298. doi: 10.5505/TurkHijyen.2017.28291

[ref26] Chisu V. Foxi C. Masala G. (2018). First molecular detection of the human pathogen *Rickettsia raoultii* and other spotted fever group rickettsiae in Ixodid ticks from wild and domestic mammals. Parasitology research, 17, pp. 3421–3429. doi: 10.1007/s00436-018-6036-y, PMID: 30078071

[ref27] Chitanga S. Chibesa K. Sichibalo K. Mubemba B. Nalubamba K. S. Muleya W.. (2021). Molecular detection and characterization of *Rickettsia* species in Ixodid ticks collected from cattle in Southern Zambia. Front. Vet. Sci. 8:684487. doi: 10.3389/fvets.2021.684487, PMID: 34164457 PMC8215536

[ref28] Chitimia-Dobler L. Schaper S. Rieß R. Bitterwolf K. Frangoulidis D. Bestehorn M.. (2019). Imported *Hyalomma* ticks in Germany in 2018. Parasit. Vectors 12, 134–139. doi: 10.1186/s13071-019-3380-4, PMID: 30909964 PMC6434826

[ref29] Djerbouh A. Kernif T. Beneldjouzi A. Socolovschi C. Kechemir N. Parola P.. (2012). The first molecular detection of *Rickettsia aeschlimannii* in the ticks of camels from Southern Algeria. Ticks Tick Borne Dis. 3, 374–376. doi: 10.1016/j.ttbdis.2012.10.014, PMID: 23168055

[ref30] De la Fuente J. Antunes S. Bonnet S. Cabezas-Cruz A. Domingos A. G. Estrada-Peña A.. (2017). Tick-pathogen interactions and vector competence: identification of molecular drivers for tick-borne diseases. Front. Cell. Infect. Microbiol. 7:114. doi: 10.3389/fcimb.2017.00114, PMID: 28439499 PMC5383669

[ref31] Demoncheaux J. P. Socolovschi C. Davoust B. Haddad S. Raoult D. Parola P. (2012). First detection of *Rickettsia aeschlimannii* in *Hyalomma dromedarii* ticks from Tunisia. Ticks Tick Borne Dis. 3, 398–402. doi: 10.1016/j.ttbdis.2012.10.003, PMID: 23182544

[ref32] de Mera I. G. F. Blanda V. Torina A. Dabaja M. F. El Romeh A. Cabezas-Cruz A.. (2018). Identification and molecular characterization of spotted fever group Rickettsiae in ticks collected from farm ruminants in Lebanon. Ticks Tick Borne Dis. 9, 104–108. doi: 10.1016/j.ttbdis.2017.10.001, PMID: 29054546

[ref33] Demir S. Erkunt Alak S. Köseoğlu A. E. Ün C. Nalçacı M. Can H. (2020). Molecular investigation of *Rickettsia* spp. and *Francisella tularensis* in ticks from three provinces of Turkey. Exp. Appl. Acarol. 81, 239–253. doi: 10.1007/s10493-020-00498-y, PMID: 32394036

[ref34] Diakou A. Norte A. C. Lopes de Carvalho I. Núncio S. Nováková M. Kautman M.. (2016). Ticks and tick-borne pathogens in wild birds in Greece. Parasitol. Res. 115, 2011–2016. doi: 10.1007/s00436-016-4943-3, PMID: 26847630

[ref35] Diarra A. Z. Almeras L. Laroche M. Berenger J. M. Koné A. K. Bocoum Z.. (2017). Molecular and MALDI-TOF identification of ticks and tick-associated bacteria in Mali. PLoS Negl. Trop. Dis. 11:e0005762. doi: 10.1371/journal.pntd.0005762, PMID: 28742123 PMC5542699

[ref36] Duscher G. G. Hodžić A. Hufnagl P. Wille-Piazzai W. Schötta A. M. Markowicz M. A.. (2018). Adult *Hyalomma marginatum* tick positive for *Rickettsia aeschlimannii* in Austria, October 2018. Eur. Secur. 23:1800595. doi: 10.2807/1560-7917.ES.2018.23.48.1800595, PMID: 30621821 PMC6280420

[ref37] Edgar R. C. (2004). MUSCLE: multiple sequence alignment with high accuracy and high throughput. Nucleic Acids Res. 32, 1792–1797. doi: 10.1016/j.sbi.2006.04.004, PMID: 15034147 PMC390337

[ref38] Ehounoud C. B. Yao K. P. Dahmani M. Achi Y. L. Amanzougaghene N. Kacou N’Douba A.. (2016). Multiple pathogens including potential new species in tick vectors in Côte d’Ivoire. PLoS Negl. Trop. Dis. 10:e0004367. doi: 10.1371/journal.pntd.0004367, PMID: 26771308 PMC4714895

[ref39] El Karkouri K. Ghigo E. Raoult D. Fournier P. E. (2022). Genomic evolution and adaptation of arthropod-associated *Rickettsia*. Sci. Rep. 12:3807. doi: 10.1038/s41598-022-07725-z, PMID: 35264613 PMC8907221

[ref40] Fernández-Soto P. Encinas Grandes A. Pérez Sánchez R. (2003). *Rickettsia aeschlimannii* in Spain: molecular evidence in *Hyalomma marginatum* and five other tick species that feed on humans. Emerg. Infect. Dis. 9, 889–890. doi: 10.3201/eid0907.030077, PMID: 12899141 PMC3023448

[ref41] Fernández-Soto P. Díaz Martín V. Pérez-Sánchez R. Encinas-Grandes A. (2009). Increased prevalence of *Rickettsia aeschlimannii* in Castilla y Leon, Spain. Eur. J. Clin. Microbiol. Infect. Dis. 28, 693–695. doi: 10.1007/s10096-008-0667-3, PMID: 18998174

[ref42] Fournier P. E. Raoult D. (2009). Current knowledge on phylogeny and taxonomy of *Rickettsia* spp. Ann. N. Y. Acad. Sci. 1166, 1–11. doi: 10.1111/j.1749-6632.2009.04528.x 19538259

[ref9008] Fournier P. E. Roux V. Raoult D. (1998). Phylogenetic analysis of spotted fever group rickettsiae by study of the outer surface protein rOmpA.. International Journal of Systematic and Evolutionary Microbiology 48, 839–849. doi: 10.1099/00207713-48-3-839 9734038

[ref43] Fournier P. E. Dumler J. S. Greub G. Zhang J. Wu Y. Raoult D. (2003). Gene sequence-based criteria for identification of new *Rickettsia* isolates and description of *Rickettsia heilongjiangensis* sp. nov. J. Clin. Microbiol. 41, 5456–5465. doi: 10.1128/JCM.41.12.5456-5465.2003, PMID: 14662925 PMC308961

[ref44] Gargili A. Palomar A. M. Midilli K. Portillo A. Kar S. Oteo J. A. (2012). *Rickettsia* species in ticks removed from humans in Istanbul, Turkey. Vector-Borne Zoonotic Dis. 12, 938–941. doi: 10.1089/vbz.2012.0996, PMID: 22925016 PMC3491622

[ref45] Germanakis A. Chochlakis D. Angelakis E. Tselentis Y. Psaroulaki A. (2013). *Rickettsia aeschlimannii* infection in a man, Greece. Emerg. Infect. Dis. 19:1176. doi: 10.3201/eid1907.130232 23764167 PMC3713992

[ref46] Getange D. Bargul J. L. Kanduma E. Collins M. Bodha B. Denge D.. (2021). Ticks and tick-borne pathogens associated with dromedary camels (*Camelus dromedarius*) in northern Kenya. Microorganisms 9:1414. doi: 10.3390/microorganisms9071414 34209060 PMC8306667

[ref47] Ghasemi A. Latifian M. Esmaeili S. Naddaf S. R. Mostafavi E. (2022). Molecular surveillance for *Rickettsia* spp. and *Bartonella* spp. in ticks from Northern Iran. PLoS One 17:e0278579. doi: 10.1371/journal.pone.0278579, PMID: 36476750 PMC9728842

[ref48] Godani S. K. Chengo M. N. Muturi M. W. (2022). Zoonotic pathogens detected in ticks in Kenyan game reserves. Adv. Entomol. 11, 1–9. doi: 10.4236/ae.2023.111001

[ref49] Grandi G. Chitimia-Dobler L. Choklikitumnuey P. Strube C. Springer A. Albihn A.. (2020). First records of adult *Hyalomma marginatum* and *H. rufipes* ticks (Acari: Ixodidae) in Sweden. Ticks Tick-borne Dis. 11:101403. doi: 10.1016/j.ttbdis.2020.101403, PMID: 32037097

[ref50] Grech-Angelini S. Stachurski F. Vayssier-Taussat M. Devillers E. Casabianca F. Lancelot R.. (2020). Tick-borne pathogens in ticks (Acari: Ixodidae) collected from various domestic and wild hosts in Corsica (France), a Mediterranean island environment. Transbound. Emerg. Dis. 67, 745–757. doi: 10.1111/tbed.13393, PMID: 31630482

[ref51] Guglielmone A.A. Robbins R.G., (2018). Hard Ticks (Acari: Ixodida: Ixodidae) Parasitizing Humans. Cham: Springer, 230

[ref52] Halajian A. Palomar A. M. Portillo A. Heyne H. Romero L. Oteo J. A. (2018). Detection of zoonotic agents and a new *Rickettsia* strain in ticks from donkeys from South Africa: implications for travel medicine. Travel Med. Infect. Dis. 26, 43–50. doi: 10.1016/j.tmaid.2018.10.007, PMID: 30312734

[ref53] Hansford K. M. Carter D. Gillingham E. L. Hernandez-Triana L. M. Chamberlain J. Cull B.. (2019). *Hyalomma rufipes* on an untraveled horse: is this the first evidence of *Hyalomma* nymphs successfully moulting in the United Kingdom? Ticks Tick-borne Dis. 10, 704–708. doi: 10.1016/j.ttbdis.2019.03.003, PMID: 30876825

[ref54] Hoogstraal H. El Kammah K.M. Santana F.J. Van Peenen P.D. (1973). Studies on southeast Asian *Haemaphysalis* ticks (Ixodoidea: Ixodidae). *H.(Kaiseriana) lagrangei* Larrousse: identity, distribution, and hosts. The Journal of Parasitology , 1118–1129. doi: 10.2307/3278651 4760643

[ref55] Hoogstraal H. Trapido H. Kohls G. M. (1996). Studies on southeast Asian *Haemaphysalis* ticks (Ixodoidea, Ixodidae). Speciation in the H.(Kaiseriana) obesa group: H. semermis Neumann, H. obesa Larrousse, H. roubaudi Toumanoff, H. montgomeryi Nuttall, and H. hirsuta sp. n.” The Journal of Parasitology 169–191. doi: 10.2307/3276410 5910451

[ref56] Hoogstraal H. Varma M. G. R. (1962). *Haemaphysalis cornupunctata* sp. n. and *H. kashmirensis* sp. n. from Kashmir, with Notes on *H. sundrai* Sharif and *H. sewelli* Sharif of India and Pakistan (Ixodoidea, Ixodidae). J. Parasitol. 48, 185–194. doi: 10.2307/3275561 13908757

[ref57] Geevarghese G. Mishra A.C., (2011). Haemaphysalis ticks of India. Waltham, MA: Elsevier.

[ref58] Igolkina Y. Rar V. Krasnova E. Filimonova E. Tikunov A. Epikhina T.. (2022). Occurrence and clinical manifestations of tick-borne rickettsioses in Western Siberia: first Russian cases of *Rickettsia aeschlimannii* and *Rickettsia slovaca* infections. Ticks Tick-borne Diseases 13:101927. doi: 10.1016/j.ttbdis.2022.101927, PMID: 35220061

[ref59] Jian Y. Li J. Adjou Moumouni P. F. Zhang X. Tumwebaze M. A. Wang G.. (2020). Human spotted fever group *Rickettsia* infecting yaks (*Bos grunniens*) in the Qinghai-Tibetan plateau area. Pathogens 9:249. doi: 10.1016/j.ttbdis.2020.101466, PMID: 32231020 PMC7238049

[ref60] Jiao J. Yu Y. He P. Wan W. OuYang X. Wen B.. (2022). First detection of *Rickettsia aeschlimannii* in *Hyalomma marginatum* in tibet, China. Zoonoses

[ref61] Kamani J. Baneth G. Apanaskevich D. A. Mumcuoglu K. Y. Harrus S. (2015). Molecular detection of *Rickettsia aeschlimannii* in *Hyalomma* spp. ticks from camels (*Camelus dromedarius*) in Nigeria, West Africa. Med. Vet. Entomol. 29, 205–209. doi: 10.1111/mve.12094, PMID: 25565180

[ref62] Karasartova D. Gureser A. S. Gokce T. Celebi B. Yapar D. Keskin A.. (2018). Bacterial and protozoal pathogens found in ticks collected from humans in Corum province of Turkey. PLoS Negl. Trop. Dis. 12:e0006395. doi: 10.1371/journal.pntd.0006395, PMID: 29649265 PMC5916866

[ref63] Karim S. Budachetri K. Mukherjee N. Williams J. Kausar A. Hassan M. J.. (2017). A study of ticks and tick-borne livestock pathogens in Pakistan. PLoS Negl. Trop. Dis. 11:e0005681. doi: 10.1371/journal.pntd.0005681, PMID: 28650978 PMC5501686

[ref64] Keskin A. Bursali A. (2016). Detection of *Rickettsia aeschlimannii* and *Rickettsia sibirica* mongolitimonae in *Hyalomma marginatum* (Acari: Ixodidae) ticks from Turkey. Acarologia 56, 533–536. doi: 10.1051/acarologia/20164140

[ref65] Keskin A. Bursali A. Keskin A. Tekin S. (2016). Molecular detection of spotted fever group Rickettsiae in ticks removed from humans in Turkey. Ticks Tick-borne Dis. 7, 951–953. doi: 10.1016/j.ttbdis.2016.04.015, PMID: 27131413

[ref66] Khan M. Islam N. Khan A. Islam Z. U. Muñoz-Leal S. Labruna M. B.. (2022). New records of *Amblyomma gervaisi* from Pakistan, with detection of a reptile-associated *Borrelia* sp. Ticks Tick-borne Dis. 13:102047. doi: 10.1016/j.ttbdis.2022.102047 36156362

[ref67] Khan S. M. Khan M. Alouffi A. Almutairi M. M. Numan M. Ullah S.. (2023). Phylogenetic position of *Haemaphysalis kashmirensis* and *Haemaphysalis cornupunctata*, with notes on *Rickettsia* spp. Genes 14:360. doi: 10.3390/genes14020360, PMID: 36833287 PMC9956137

[ref68] Khan Z. Shehla S. Alouffi A. Kashif Obaid M. Zeb Khan A. Almutairi M. M.. (2022). Molecular survey and genetic characterization of *Anaplasma marginale* in ticks collected from livestock hosts in Pakistan. Animals 12:1708. doi: 10.3390/ani12131708, PMID: 35804607 PMC9264954

[ref69] Khrouf F. M'Ghirbi Y. Znazen A. Ben Jemaa M. Hammami A. Bouattour A. (2014). Detection of *Rickettsia* in *Rhipicephalus sanguineus* ticks and *Ctenocephalides felis* fleas from southeastern Tunisia by reverse line blot assay. J. Clin. Microbiol. 52, 268–274. doi: 10.1128/JCM.01925-13 24226919 PMC3911462

[ref70] Kleinerman G. Baneth G. Mumcuoglu K. Y. van Straten M. Berlin D. Apanaskevich D. A.. (2013). Molecular detection of *Rickettsia africae*, *Rickettsia aeschlimannii*, and *Rickettsia sibirica* mongolitimonae in camels and *Hyalomma* spp. ticks from Israel. Vector Borne Zoonotic Dis 13, 851–856. doi: 10.1089/vbz.2013.1330, PMID: 24107206

[ref71] Koczwarska J. Pawełczyk A. Dunaj-Małyszko J. Polaczyk J. Welc-Falęciak R. (2023). *Rickettsia* species in *Dermacentor reticulatus* ticks feeding on human skin and clinical manifestations of tick-borne infections after tick bite. Sci. Rep. 13:9930. doi: 10.1038/s41598-023-37059-3, PMID: 37336983 PMC10279655

[ref72] Koka H. Sang R. Kutima H. L. Musila L. (2017). The detection of spotted fever group *Rickettsia* DNA in tick samples from pastoral communities in Kenya. J. Med. Entomol. 54, 774–780. doi: 10.1093/jme/tjw238, PMID: 28073909 PMC5850802

[ref73] Kratou M. Belkahia H. Selmi R. Andolsi R. Dhibi M. Mhadhbi M.. (2023). Diversity and phylogeny of cattle ixodid ticks and associated spotted fever group *Rickettsia* spp. in Tunisia. Pathogens 12:552. doi: 10.3390/pathogens12040552, PMID: 37111438 PMC10146803

[ref74] Kumar S. Stecher G. Li M. Knyaz C. Tamura K. (2018). MEGA X: molecular evolutionary genetics analysis across computing platforms. Mol. Biol. Evol. 35:1547. doi: 10.1093/molbev/msy096/4990887 29722887 PMC5967553

[ref76] Labruna M. B. (2009). Ecology of rickettsia in South America. Ann. N. Y. Acad. Sci. 1166, 156–166. doi: 10.1111/j.1749-6632.2009.04516.x 19538276

[ref77] Labruna M. B. Whitworth T. Horta M. C. Bouyer D. H. McBride J. W. Pinter A.. (2004). *Rickettsia* species infecting *Amblyomma cooperi* ticks from an area in the state of São Paulo, Brazil, where Brazilian spotted fever is endemic. J. Clin. Microbiol. 42, 90–98. doi: 10.1128/JCM.42.1.90-98.2004, PMID: 14715737 PMC321730

[ref78] Leulmi H. Aouadi A. Bitam I. Bessas A. Benakhla A. Raoult D.. (2016). Detection of *Bartonella tamiae*, *Coxiella burnetii* and Rickettsiae in arthropods and tissues from wild and domestic animals in northeastern Algeria. Parasit. Vectors 9, 1–8. doi: 10.1186/s13071-016-1316-9, PMID: 26791781 PMC4721140

[ref79] Loftis A. D. Reeves W. K. Szumlas D. E. Abbassy M. M. Helmy I. M. Moriarity J. R.. (2006). Rickettsial agents in Egyptian ticks collected from domestic animals. Exp. Appl. Acarol. 40, 67–81. doi: 10.1007/s10493-006-9025-2, PMID: 17004028

[ref81] Maioli G. Bonilauri P. Barbieri I. Calzolari M. Francesco D.F. And Dottori M., Detection of spotted fever group *Rickettsia* and *Anaplasma phagocytophilum* in ticks removed from wild animals in northern Italy in 2009 (2009).

[ref82] Mancini F. Ciccozzi M. Lo Presti A. Cella E. Giovanetti M. Di Luca M.. (2015). Characterization of spotted fever group Rickettsiae in ticks from a city park of Rome, Italy. Ann. Ist. Super Sanita 51, 284–290. doi: 10.4415/ANN_15_04_07, PMID: 26783214

[ref83] Matsumoto K. Parola P. Brouqui P. Raoult D. (2004). *Rickettsia aeschlimannii* in *Hyalomma* ticks from Corsica. Eur. J. Clin. Microbiol. Infect. Dis. 23, 732–734. doi: 10.13140/RG.2.2.22971.98080, PMID: 15309667

[ref84] McGinley L. Hansford K. M. Cull B. Gillingham E. L. Carter D. P. Chamberlain J. F.. (2021). First report of human exposure to *Hyalomma marginatum* in England: further evidence of a *Hyalomma* moulting event in North-Western Europe? Ticks and Tick-borne Diseases 12:101541. doi: 10.1016/j.ttbdis.2020.101541, PMID: 33007668

[ref85] Mediannikov O. Diatta G. Fenollar F. Sokhna C. Trape J. F. Raoult D. (2010). Tick-borne rickettsioses, neglected emerging diseases in rural Senegal. PLoS Negl. Trop. Dis. 4:e821. doi: 10.1371/journal.pntd.0000821, PMID: 20856858 PMC2939048

[ref86] Mokrani N. Parola P. Tebbal S. Dalichaouche M. Aouati A. Raoult D. (2008). *Rickettsia aeschlimannii* infection, Algeria. Emerg. Infect. Dis. 14, 1814–1815. doi: 10.3201/eid1411.071221, PMID: 18976583 PMC2630723

[ref87] Morita C. El Hussein A. R. M. Matsuda E. Abdel Gabbar K. M. A. Muramatsu Y. Abdel Rahman M. B.. (2004). Spotted fever group Rickettsiae from ticks captured in Sudan. Jpn. J. Infect. Dis. 57, 107–109. PMID: 15218219

[ref89] Mumcuoglu K. Y. Arslan-Akveran G. Aydogdu S. Karasartova D. Koşar A. Savci U.. (2022). Pathogens in ticks collected in Israel: II. Bacteria and protozoa found in *Rhipicephalus sanguineus* sensu lato and *Rhipicephalus turanicus*. Ticks Tick-borne Dis. 13:101986. doi: 10.1016/j.ttbdis.2022.101986, PMID: 35816829

[ref90] Mura A. Masala G. Tola S. Satta G. Fois F. Piras P.. (2008a). First direct detection of rickettsial pathogens and a new rickettsia, ‘*Candidatus* Rickettsia barbariae’, in ticks from Sardinia, Italy. Clin. Microbiol. Infect. 14, 1028–1033. doi: 10.1111/j.1469-0691.2008.02082.x 19040474

[ref91] Mura A. Socolovschi C. Ginesta J. Lafrance B. Magnan S. Rolain J. M.. (2008b). Molecular detection of spotted fever group rickettsiae in ticks from Ethiopia and Chad. Trans. R. Soc. Trop. Med. Hyg. 102, 945–949. doi: 10.1016/j.trstmh.2008.03.015, PMID: 18440576

[ref92] Mutai B. K. Wainaina J. M. Magiri C. G. Nganga J. K. Ithondeka P. M. Njagi O. N.. (2013). Zoonotic surveillance for Rickettsiae in domestic animals in Kenya. Vector Borne Zoonotic Dis. 13, 360–366. doi: 10.1089/vbz.2012.0977, PMID: 23477290

[ref93] Nader J. Król N. Pfeffer M. Ohlendorf V. Marklewitz M. Drosten C.. (2018). The diversity of tick-borne bacteria and parasites in ticks collected from the Strandja Nature Park in South-Eastern Bulgaria. Parasit. Vectors 11, 165–110. doi: 10.1186/s13071-018-2721-z, PMID: 29554928 PMC5859726

[ref94] Numan M. Islam N. Adnan M. Zaman Safi S. Chitimia-Dobler L. Labruna M. B.. (2022). First genetic report of *Ixodes kashmiricus* and associated *Rickettsia* sp. Parasit. Vectors 15, 378–312. doi: 10.1186/s13071-022-05509-y, PMID: 36261834 PMC9583563

[ref95] Omondi D. Masiga D. K. Fielding B. C. Kariuki E. Ajamma Y. U. Mwamuye M. M.. (2017). Molecular detection of tick-borne pathogen diversities in ticks from livestock and reptiles along the shores and adjacent islands of Lake Victoria and Lake Baringo, Kenya. Front. Vet. Sci. 4:73. doi: 10.3389/fvets.2017.00073, PMID: 28620610 PMC5451513

[ref96] Onyiche T. E. Răileanu C. Tauchmann O. Fischer S. Vasić A. Schäfer M.. (2020). Prevalence and molecular characterization of ticks and tick-borne pathogens of one-humped camels (*Camelus dromedarius*) in Nigeria. Parasit. Vectors 13, 428–416. doi: 10.1186/s13071-020-04272-2, PMID: 32838795 PMC7445909

[ref97] Orkun Ö. Çakmak A. (2019, 2019). Molecular identification of tick-borne bacteria in wild animals and their ticks in Central Anatolia, Turkey. Comp. Immunol. Microbiol. Infect. Dis. 63, 58–65. doi: 10.1016/j.cimid.2018.12.007, PMID: 30961819

[ref98] Orkun Ö. Karaer Z. Çakmak A. Nalbantoğlu S. (2014). Identification of tick-borne pathogens in ticks feeding on humans in Turkey. PLoS Negl. Trop. Dis. 8:e3067. doi: 10.1371/journal.pntd.0003067, PMID: 25101999 PMC4125308

[ref99] Oteo J. A. Portillo A. Blanco J. R. Ibarra V. Pérez-Martínez L. Izco C.. (2005). Low risk of developing human *Rickettsia aeschlimannii* infection in the north of Spain. Ann. N. Y. Acad. Sci. 1063, 349–351. doi: 10.1196/annals.1355.057 16481540

[ref100] Otranto D. Dantas-Torres F. Giannelli A. Latrofa M. S. Cascio A. Cazzin S.. (2014). Ticks infesting humans in Italy and associated pathogens. Parasit. Vectors 7, 1–9. doi: 10.1186/1756-3305-7-3, PMID: 25023709 PMC4223688

[ref101] Paguem A. Manchang K. Kamtsap P. Renz A. Schaper S. Dobler G.. (2023). Ticks and rickettsiae associated with wild animals sold in bush meat markets in Cameroon. Pathogens 12:348. doi: 10.3390/pathogens12020348, PMID: 36839620 PMC9964434

[ref102] Palomar A. M. Molina I. Bocanegra C. Portillo A. Salvador F. Moreno M.. (2022). Old zoonotic agents and novel variants of tick-borne microorganisms from Benguela (Angola), July 2017. Parasit. Vectors 15:140. doi: 10.1186/s13071-022-05238-2, PMID: 35449022 PMC9022410

[ref103] Palomar A. M. Portillo A. Mazuelas D. Roncero L. Arizaga J. Crespo A.. (2016). Molecular analysis of Crimean-Congo hemorrhagic fever virus and *Rickettsia* in *Hyalomma marginatum* ticks removed from patients (Spain) and birds (Spain and Morocco), 2009–2015. Ticks Tick-borne Dis. 7, 983–987. doi: 10.1016/j.ttbdis.2016.05.004, PMID: 27215620

[ref104] Parola P. Inokuma H. Camicas J. L. Brouqui P. Raoult D. (2001). Detection and identification of spotted fever group Rickettsiae and Ehrlichiae in African ticks. Emerg. Infect. Dis. 7, 1014–1017. doi: 10.3201/eid0706.010616, PMID: 11747731 PMC2631901

[ref105] Parola P. Paddock C. D. Socolovschi C. Labruna M. B. Mediannikov O. Kernif T.. (2013). Update on tick-borne rickettsioses around the world: a geographic approach. Clin. Microbiol. Rev. 26, 657–702. doi: 10.1128/CMR.00032-13, PMID: 24092850 PMC3811236

[ref106] Pascucci I. Di Domenico M. Curini V. Cocco A. Averaimo D. D’Alterio N.. (2019a). Diversity of *Rickettsia* in ticks collected in Abruzzi and Molise regions (Central Italy). Microorganisms 7:696. doi: 10.3390/microorganisms7120696, PMID: 31847276 PMC6956140

[ref107] Pascucci I. Di Domenico M. Dondona G. C. Di Gennaro A. Polci A. Dondona A. C.. (2019b). Assessing the role of migratory birds in the introduction of ticks and tick-borne pathogens from African countries: an Italian experience. Ticks Tick-borne Dis. 10:101272. doi: 10.1016/j.ttbdis.2019.101272, PMID: 31481344

[ref108] Portillo A. Santibáñez P. Santibáñez S. Pérez-Martínez L. Oteo J. A. (2008). Detection of Rickettsia spp. in Haemaphysalis ticks collected in La Rioja, Spain. Vector-Borne Zoonotic Dis. 8, 653–658. doi: 10.1089/vbz.2007.0272, PMID: 18454590

[ref109] Pretorius A. M. Birtles R. J. (2002). *Rickettsia aeschlimannii*: a new pathogenic spotted fever group *Rickettsia*, South Africa. Emerg. Infect. Dis. 8, 874a–8874a. doi: 10.3201/eid0808.020199 PMC2732514 12141981

[ref110] Psaroulaki A. Ragiadakou D. Kouris G. Papadopoulos B. Chaniotis B. Tselentis Y. (2006). Ticks, tick-borne Rickettsiae, and *Coxiella burnetii* in the Greek island of Cephalonia. Ann. N. Y. Acad. Sci. 1078, 389–399. doi: 10.1196/annals.1374.077 17114747

[ref111] Punda-Polic V. Petrovec M. Trilar T. Duh D. Bradaric N. Klismanic Z.. (2003). “Detection and identification of spotted fever group Rickettsiae in ticks collected in southern Croatia” in Ticks and tick-borne pathogens: Proceedings of the 4th international conference on ticks and tick-borne pathogens the Banff Centre Banff, Alberta, Canada 21–26 July 2002 (Dordrecht: Springer Netherlands), 169–176. 10.1023/a:1025334113190 14570128

[ref112] Qiu Y. Simuunza M. Kajihara M. Ndebe J. Saasa N. Kapila P.. (2022). Detection of tick-borne bacterial and protozoan pathogens in ticks from the Zambia-Angola border. Pathogens 11:566. doi: 10.3390/pathogens11050566, PMID: 35631087 PMC9144998

[ref113] Raoult D. Fournier P. E. Abboud P. Caron F. (2002). First documented human *Rickettsia aeschlimannii* infection. Emerg. Infect. Dis. 8, 748–749. doi: 10.3201/eid0807.010480, PMID: 12095451 PMC2730330

[ref114] Regnery R. L. Spruill C. L. Plikaytis B. (1991). Genotypic identification of rickettsiae and estimation of intraspecies sequence divergence for portions of two rickettsial genes. J. Bacteriol. 173, 1576–1589. doi: 10.1128/jb.173.5.1576-1589.1991, PMID: 1671856 PMC207306

[ref115] Rollins R. E. Schaper S. Kahlhofer C. Frangoulidis D. Strauß A. F. Cardinale M.. (2021). Ticks (Acari: Ixodidae) on birds migrating to the island of Ponza, Italy, and the tick-borne pathogens they carry. Ticks Tick-borne Dis. 12:101590. doi: 10.1016/j.ttbdis.2020.101590, PMID: 33113477

[ref116] Roux V. Raoult D. (2000). Phylogenetic analysis of members of the genus *Rickettsia* using the gene encoding the outer-membrane protein rOmpB (ompB). Int. J. Syst. Evol. Microbiol. 50, 1449–1455. doi: 10.1099/00207713-50-4-1449, PMID: 10939649

[ref117] Roux V. Fournier P. E. Raoult D. (1997). Differentiation of spotted fever group Rickettsiae by sequencing and analysis of restriction fragment length polymorphism of PCR-amplified DNA of the gene encoding the protein rOmpA. J. Clin. Microbiol. 34, 2058–2065. doi: 10.1128/JCM.34.9.2058-2065.1996 PMC229190 8862558

[ref118] Rumer L. Graser E. Hillebrand T. Talaska T. Dautel H. Mediannikov O.. (2011). *Rickettsia aeschlimannii* in *Hyalomma marginatum* ticks, Germany. Emerging infectious diseases 17, 325–326. doi: 10.3201/eid1702.100308, PMID: 21291625 PMC3204748

[ref119] Sadeddine R. Diarra A. Z. Laroche M. Mediannikov O. Righi S. Benakhla A.. (2020). Molecular identification of protozoal and bacterial organisms in domestic animals and their infesting ticks from North-Eastern Algeria. Ticks Tick-borne Dis. 11:101330. doi: 10.1016/j.ttbdis.2019.101330, PMID: 31786146

[ref120] Saman E. A. G. Chegeni A. H. (2022). Hard ticks (Acari: Ixodidae) infected by Rickettsias: the first record of *Rickettsia aeschlimannii* (Rickettsiales: Rickettsiaceae) in Iran. Syst. Appl. Acarol. 27, 749–762. doi: 10.11158/saa.27.4.7

[ref121] Sambou M. Faye N. Bassène H. Diatta G. Raoult D. Mediannikov O. (2014). Identification of Rickettsial pathogens in ixodid ticks in northern Senegal. Ticks Tick-borne Dis. 5, 552–556. doi: 10.1016/j.ttbdis.2014.04.002, PMID: 24908548

[ref122] Sambrook J. Fritsch E.F. Maniatis T. Molecular cloning: a laboratory manual, 2nd; Cold Spring Harbor Laboratory Press: New York, NY, USA, (1989)

[ref123] Santos-Silva M. M. Sousa R. Santos A. S. Melo P. Encarnação V. Bacellar F. (2006). Ticks parasitizing wild birds in Portugal: detection of *Rickettsia aeschlimannii*, *R. helvetica* and *R. massiliae*. Exp. Appl. Acarol. 39, 331–338. doi: 10.1007/s10493-006-9008-3, PMID: 16897568

[ref124] Sarih M. Socolovschi C. Boudebouch N. Hassar M. Raoult D. Parola P. (2008). Spotted fever group Rickettsiae in ticks, Morocco. Emerg. Infect. Dis. 14, 1067–1073. doi: 10.3201/eid1407.070096, PMID: 18598627 PMC2600325

[ref125] Satjanadumrong J. Robinson M. T. Hughes T. Blacksell S. D. (2019). Distribution and ecological drivers of spotted fever group *Rickettsia* in Asia. EcoHealth 16, 611–626. doi: 10.1007/s10393-019-01409-3, PMID: 30993545 PMC6910891

[ref126] Scarpulla M. Barlozzari G. Marcario A. Salvato L. Blanda V. De Liberato C.. (2016). Molecular detection and characterization of spotted fever group Rickettsiae in ticks from Central Italy. Ticks Tick-borne Dis. 7, 1052–1056. doi: 10.1016/j.ttbdis.2016.06.003, PMID: 27365155

[ref127] Selmi R. Ben Said M. Ben Yahia H. Abdelaali H. Messadi L. (2020). Molecular epidemiology and phylogeny of spotted fever group *Rickettsia* in camels (*Camelus dromedarius*) and their infesting ticks from Tunisia. Transbound. Emerg. Dis. 67, 733–744. doi: 10.1111/tbed.13392, PMID: 31626722

[ref128] Sgroi G. Iatta R. Lia R. P. Napoli E. Buono F. Bezerra-Santos M. A.. (2022). Tick exposure and risk of tick-borne pathogens infection in hunters and hunting dogs: a citizen science approach. Transbound. Emerg. Dis. 69, e386–e393. doi: 10.1111/tbed.14314, PMID: 34487635 PMC9546254

[ref129] Shehla S. Ullah F. Alouffi A. Almutairi M. M. Khan Z. Tanaka T.. (2023). Association of SFG *Rickettsia massiliae* and *Candidatus* Rickettsia shennongii with different hard ticks infesting livestock hosts. Pathogens 12:1080. doi: 10.3390/pathogens12091080, PMID: 37764888 PMC10536372

[ref130] Shpynov S. Fournier P. E. Rudakov N. Tankibaev M. Tarasevich I. Raoult D. (2004). Detection of a rickettsia closely related to *Rickettsia aeschlimannii*, “*Rickettsia heilongjiangensis*,” *Rickettsia* sp. strain RpA4, and *Ehrlichia muris* in ticks collected in Russia and Kazakhstan. J. Clin. Microbiol. 42, 2221–2223. doi: 10.1128/JCM.42.5.2221-2223.2004, PMID: 15131195 PMC404608

[ref131] Shpynov S. Rudakov N. Tohkov Y. Matushchenkso A. Tarasevich I. Raoult D.. (2009). Detection of *Rickettsia aeschlimannii* in *Hyalomma marginatum* ticks in western Russia. Clin. Microbiol. Infect. 15, 315–316. doi: 10.1111/j.1469-0691.2008.02256.x, PMID: 19438620

[ref132] Shuaib Y. A. Elhag A. M. A. W. Brima Y. A. Abdalla M. A. Bakiet A. O. Mohmed-Noor S. E. T.. (2020). Ixodid tick species and two tick-borne pathogens in three areas in the Sudan. Parasitol. Res. 119, 385–394. doi: 10.1007/s00436-019-06458-9, PMID: 31901105

[ref133] Socolovschi C. Mediannikov O. Raoult D. Parola P. (2009). The relationship between spotted fever group Rickettsiae and ixodid ticks. Vet. Res. 40:34. doi: 10.1051/vetres/2009017, PMID: 19358804 PMC2695030

[ref134] Socolovschi C. Reynaud P. Raoult D. Parola P. (2011). “Rickettsiae and Borrelia in ticks on migratory birds from the Camargue National Park, France” in Abstract book of the 6th international meeting on Rickettsiae and Rickettsial diseases (Heraklion, Crete, Greece)

[ref135] Song S. Chen C. Yang M. Zhao S. Wang B. Hornok S.. (2018). Diversity of *Rickettsia* species in border regions of northwestern China. Parasit. Vectors 11, 634–637. doi: 10.1186/s13071-018-3233-6, PMID: 30545379 PMC6293579

[ref136] Springer A. Shuaib Y. A. Isaa M. H. Ezz-Eldin M. I. E. Osman A. Y. Yagoub I. A.. (2020). Tick fauna and associated *Rickettsia*, *Theileria*, and *Babesia* spp. in domestic animals in Sudan (North Kordofan and Kassala States). Microorganisms 8:1969. doi: 10.3390/microorganisms8121969, PMID: 33322349 PMC7763929

[ref137] Sukhiashvili R. Zhgenti E. Khmaladze E. Burjanadze I. Imnadze P. Jiang J.. (2020). Identification and distribution of nine tick-borne spotted fever group Rickettsiae in the country of Georgia. Ticks Tick-borne Dis. 11:101470. doi: 10.1016/j.ttbdis.2020.101470, PMID: 32723640

[ref138] Thompson J. D. Higgins D. G. Gibson T. J. (1994). CLUSTAL W: improving the sensitivity of progressive multiple sequence alignment through sequence weighting, position-specific gap penalties and weight matrix choice. Nucleic Acids Res. 22, 4673–4680. doi: 10.1093/nar/22.22.4673, PMID: 7984417 PMC308517

[ref7] Tila H. Khan M. Almutairi M. M. Alouffi A. Ahmed H. Tanaka T.. (2023). First report on detection of Hepatozoon ayorgbor in *Rhipicephalus haemaphysaloides* and *Hepatozoon colubri* in *Haemaphysalis sulcata* and *Hyalomma anatolicum*: risks of spillover of *Hepatozoon* spp. from wildlife to domestic animals. Front. Vet. Sci. 10:1255482. doi: 10.3389/fvets.2023.1255482, PMID: 37789871 PMC10544907

[ref139] Toma L. Mancini F. Di Luca M. Cecere J. G. Bianchi R. Khoury C.. (2014). Detection of microbial agents in ticks collected from migratory birds in Central Italy. Vector-borne Zoonotic Dis. 14, 199–205. doi: 10.1089/vbz.2013.1458, PMID: 24576218 PMC3952585

[ref140] Tomassone L. Conte V. Parrilla G. De Meneghi D. (2010). Rickettsia infection in dogs and *Rickettsia parkeri* in *Amblyomma tigrinum* ticks, Cochabamba department, Bolivia. Vector-Borne and Zoonotic Diseases 10, 953–958. doi: 10.1089/vbz.2009.0126, PMID: 20426684

[ref141] Tomassone L. De Meneghi D. Adakal H. Rodighiero P. Pressi G. Grego E. (2016). Detection of *Rickettsia aeschlimannii* and *Rickettsia africae* in ixodid ticks from Burkina Faso and Somali Region of Ethiopia by new real-time PCR assays. Ticks Tick-borne Dis. 7, 1082–1088. doi: 10.1016/j.ttbdis.2016.09.005, PMID: 27641952

[ref142] Tomassone L. Grego E. Auricchio D. Iori A. Giannini F. Rambozzi L. (2013). Lyme borreliosis spirochetes and spotted fever group Rickettsiae in ixodid ticks from Pianosa island, Tuscany archipelago, Italy. Vector Borne Zoonotic Dis. 13, 84–91. doi: 10.1089/vbz.2012.1046, PMID: 23289398

[ref143] Tosoni A. Mirijello A. Ciervo A. Mancini F. Rezza G. Damiano F.. (2016). Human *Rickettsia aeschlimannii* infection: first case with acute hepatitis and review. Eur. Rev. Med. Pharmacol. Sci. 20, 2630–2633. PMID: 27383315

[ref144] Ullah S. Alouffi A. Almutairi M. M. Islam N. Rehman G. Ul Islam Z.. (2023). First report of *Rickettsia conorii* in *Hyalomma kumari* ticks. Animals 13:1488. doi: 10.3390/ani13091488, PMID: 37174525 PMC10177544

[ref145] Vanegas A. Keller C. Krüger A. Manchang T. K. Hagen R. M. Frickmann H.. (2018). Molecular detection of spotted fever group Rickettsiae in ticks from Cameroon. Ticks Tick Borne Dis. 9, 1049–1056. doi: 10.1016/j.ttbdis.2018.03.022, PMID: 29636236

[ref146] Walker J.B. Keirans J.E. Horak I.G. (2000) The genus *Rhipicephalus* (Acari, Ixodidae): a guide to the brown ticks of the world. Cambridge, NY Cambridge University Press

[ref147] Koponen S. Niemi R. (2000). Review: Brown ticks of the world. Entomol. Fennica 11:124. doi: 10.33338/ef.84056

[ref148] Wallmenius K. Barboutis C. Fransson T. Jaenson T. G. Lindgren P. E. Nyström F.. (2014). Spotted fever *Rickettsia* species in *Hyalomma* and Ixodes ticks infesting migratory birds in the European Mediterranean area. Parasit. Vectors 7, 1–20. doi: 10.1186/1756-3305-7-318, PMID: 25011617 PMC4230250

[ref149] Wei Q. Q. Guo L. P. Wang A. D. Mu L. M. Zhang K. Chen C. F.. (2015). The first detection of *Rickettsia aeschlimannii* and *Rickettsia massiliae* in *Rhipicephalus turanicus* ticks, in Northwest China. Parasit. Vectors 8, 631–634. doi: 10.1186/s13071-015-1242-2, PMID: 26652857 PMC4675064

[ref150] Yessinou R. E. Cazan C. D. Panait L. C. Mollong E. Biguezoton A. S. Bonnet S. I.. (2023). New geographical records for tick-borne pathogens in ticks collected from cattle in Benin and Togo. Vet. Med. Sci. 9, 345–352. doi: 10.1002/vms3.1022, PMID: 36508582 PMC9856996

[ref151] Znazen A. Rolain J. M. Hammami N. Hammami A. Ben Jemaa M. Raoult D. (2006). *Rickettsia felis* infection, Tunisia. Emerg. Infect. Dis. 12, 138–140. doi: 10.3201/eid1201.050876, PMID: 16494731 PMC3291393

